# MeCP2 duplication causes hyperandrogenism by upregulating LHCGR and downregulating RORα

**DOI:** 10.1038/s41419-021-04277-4

**Published:** 2021-10-25

**Authors:** Yu-Meng Wang, Yu Wu, Yu-Fang Zheng, Hong-Yan Wang

**Affiliations:** 1 Obstetrics and Gynaecology Hospital, State Key Laboratory of Genetic Engineering at School of Life Sciences, Institute of Reproduction and Development, Fudan University, Shanghai, 200011 China; 2 Institute of Developmental Biology & Molecular Medicine, Fudan University, Shanghai, 200433 China; 3 Children’s Hospital of Fudan University, 399 Wanyuan Road, Shanghai, 201102 China

**Keywords:** Metabolism, Metabolic disorders

## Abstract

Duplication of *MECP2* (methyl-CpG-binding protein 2) gene causes a serious neurological and developmental disorder called *MECP2* duplication syndrome (MDS), which is usually found in males. A previous clinical study reported that MDS patient has precocious puberty with hyperandrogenism, suggesting increased MeCP2 may cause male hyperandrogenism. Here we use an MDS mouse model and confirm that *MECP2* duplication significantly upregulates androgen levels. We show for the first time that MeCP2 is highly expressed in the Leydig cells of testis, where androgen is synthesized. Mechanistically, *MECP2* duplication increases androgen synthesis and decreases androgen to estrogen conversion through either the upregulation of luteinizing hormone receptor (LHCGR) in testis, as a result of MeCP2 binds to G-quadruplex structure of *Lhcgr* promoter and recruits the transcription activator CREB1 or the downregulation of the expression of aromatase in testis by binding the CpG island of *Rorα*, an upstream regulator of aromatase. Taken together, we demonstrate that MeCP2 plays an important role in androgen synthesis, supporting a novel non-CNS function of MeCP2 in the process of sex hormone synthesis.

## Introduction

As an epigenetic regulator, the DNA binding protein methyl-CpG-binding protein 2 (MeCP2) is known for its important neurology and brain function. Loss and gain of function of MeCP2 lead to Rett syndrome (RTT) and *MECP2* duplication syndrome (MDS), respectively, which are two severe neurological disorders characterized by intellectual disability, autism, and developmental regression [1, 2]. Accordingly, the functional studies of MeCP2 have been largely focused on its role in brain development [3]. However, MeCP2 is also expressed in many other tissues, and its malfunction can lead to multiple organ anomalies [4, 5], such as pneumonia [6] and heart failure [7]. Recent studies have shown that precocious puberty with hyperandrogenism is another clinical feature of patients with MDS and MeCP2 mutations [8–10], suggesting that MeCP2 may play an important role in androgen synthesis. However, whether or how MeCP2 plays a role in the process of sex hormone synthesis is unclear.

Androgen synthesis is regulated by the pulsatile release of hypothalamic gonadotropin-releasing hormone (GnRH), which stimulates the release of luteinizing hormone (LH) into the general circulation [11]. LH binds to its receptor LHCGR (luteinizing hormone receptor) at Leydig cells and triggers a cellular response mediated by second messengers (cAMP) to increase the expression of the steroidogenic acute regulatory protein (StAR). StAR promotes the transfer of cholesterol to the inner mitochondrial membrane and initiates steroidogenesis, which is the rate-limiting step of steroidogenesis [12]. After being transferred into mitochondria, cholesterol is converted to testosterone by the action of several enzymes [13]. At last, testosterone is converted into estrogen by aromatase which is encoded by the *CYP19A1* gene [13, 14]. The epigenetic regulations of key steroidogenic enzymes and regulators are important for androgen levels. For example, hypomethylation of the promoter region and subsequent activation of the *LHCGR* gene is a potential mechanism underlying the susceptibility of polycystic ovary syndrome (PCOS) [15], which is the most common endocrine disorder in reproductive-aged women and characterized by hyperandrogenism.

In this work, we investigated how MeCP2 influences sex hormone synthesis in MeCP2^Tg1^ mice, which is a model for MDS as they are containing an extra copy of human *MECP2* [16]. We found that the level of testosterone was indeed elevated in the adolescence and adulthood MeCP2^Tg1^ mice. MeCP2 is relatively highly expressed in Leydig cells of testis, where sex hormone is synthesized. *MECP2* duplication is sufficient to cause sex hormone synthesis anomalies, primarily through upregulation of LHCGR expression and downregulation of androgen-to-estrogen conversion. Furthermore, our work reveals a novel mechanism that MeCP2 binds to the G-quadruplex structure and upregulates gene expression by recruiting the transcription activator CREB1.

## Results

### Elevated androgen in MeCP2^Tg1^ mice

**Fig. 1:**
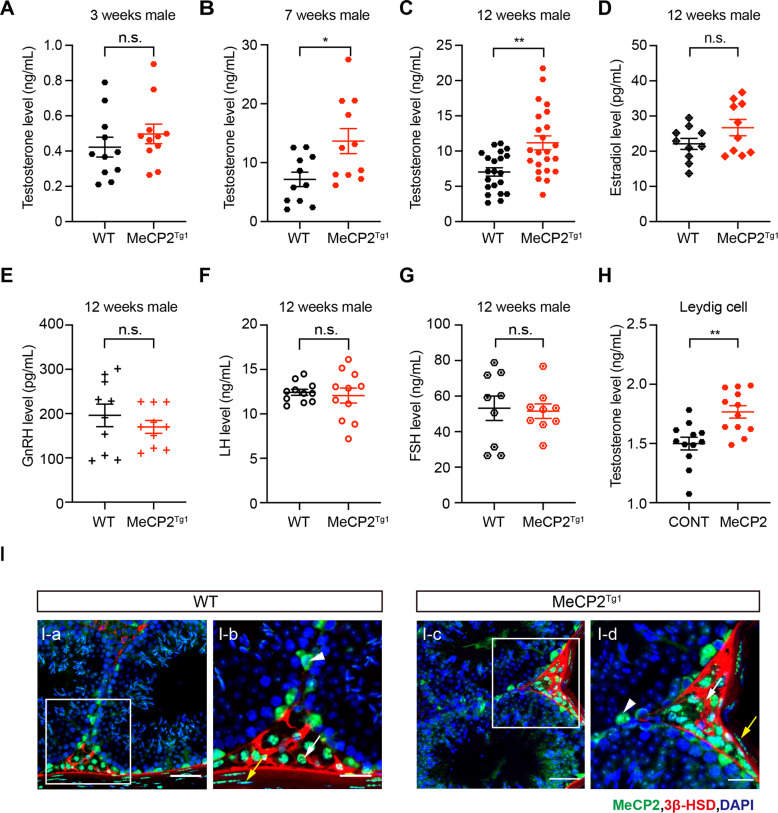
Elevated androgen in MeCP2^Tg1^ mice. **A–C** Quantification of the serum testosterone concentrations in 3-week-old (childhood) (**A**), 7-week-old (puberty) (**B**), and 12-week-old (adult) (**C**) male MeCP2^Tg1^ mice (*N* = 11, 11 and 21, respectively) and male WT littermates (*N* = 11, 11 and 23, respectively). **D** Quantification of the serum estradiol concentrations in male MeCP2^Tg1^ mice and male WT littermates. *N* = 10 for each group. **E–G** Quantification of the concentrations of the serum estradiol gonadotrophin-releasing hormones (GnRH) (**E**), luteinizing hormone (LH) (**F**) and follicle-stimulating hormone (FSH) (**G**) in 12-week-old male MeCP2^Tg1^ mice and male WT littermates. *N* ≥ 9 for each group. **H** Quantification of the testosterone concentrations in Leydig cells transfected with control vector or MeCP2-expressed plasmid. *N* = 12 for each group. **I** Immunofluorescent staining of testis sections from 3-month-old WT (I-a, I-b) and MeCP2^Tg1^ (I-c, I-d) mice with Leydig cell marker 3β-HSD (red), MeCP2 (green) and DAPI (blue). The Leydig cells (white arrows), Sertoli cells (arrowhead), and fibroblasts (yellow arrows) express MeCP2 in both WT and MeCP2^Tg1^ mice testis. Scale bars: 50 μm (I-a, I-c), 25 μm (I-b, I-d). For **A**–**H**, data are presented as mean ± s.e.m. *p < 0.05; **p < 0.01; ***p < 0.001, n.s. stands for not significant. Statistical analyses were performed using a two-tailed unpaired Student’s *t*-test.

To evaluate the correlation of elevated MeCP2 and sex hormones level in MeCP2^Tg1^ mice, the concentrations of serum testosterone were detected by ELISA in MeCP2^Tg1^ male mice or wild-type (WT) littermates males at 3-week-old (childhood), 7-week-old (puberty), and 12-week-old (adult), respectively. The testosterone level in MeCP2^Tg1^ mice was significantly higher than that in WT mice after the puberty stage (Fig. 1A–C). In 7 and 12 weeks old MeCP2^Tg1^ mice, the testosterone levels were about 2-fold higher than that in the WT littermates. The serum concentration of estradiol (E2) was also examined, but there was no significant difference between MeCP2^Tg1^ mice and WT littermates (Fig. 1D).

As MeCP2 plays a critical role in neural development, we wondered whether MeCP2 duplication related androgen elevation is impaired by the hypothalamic-pituitary axis (HPA). The levels of different hormones, including gonadotrophin-releasing hormones (GnRH), follicle-stimulating hormone (FSH), and luteinizing hormone (LH), were examined in 12-week-old mice. We found that the serum concentrations of GnRH, FSH and LH show no significant difference between MeCP2^Tg1^ mice and WT littermates (Fig. 1E–G). Also, the *Gnrh1* and *Lhb* mRNAs encoding the corresponding hormones and the receptor of GnRH in MeCP2^Tg1^ mice also showed no significant changes when compared with WT littermates (Fig. S1). These results revealed that MeCP2 duplication-induced androgen elevation is not affected by HPA. In order to evaluate the potential effect of duplicated MeCP2 on androgen synthesis, we then investigated whether MeCP2 is expressed in testis. Immunofluorescent staining on testis sections from both 3-month-old WT and MeCP2^Tg1^ male mice showed that MeCP2 is highly expressed in the 3β-HSD (Leydig cell marker) positive cells (Fig. 1I), in which testosterone (T) is produced in the presence of the luteinizing hormone (LH) [17], and the Sertoli cells, fibroblasts, and some spermatocytes are also MeCP2 positive. To further verify whether MeCP2 could upregulate androgen synthesis in testis, we overexpressed MeCP2 in TM3 cells, the Leydig cell line derived from 11–13 day mouse testis. In TM3 cells, the testosterone level was also significantly upregulated by MeCP2 overexpression (Fig. 1H), which is similar to that in MeCP2^Tg1^ mice. Taken together, the results indicated that MeCP2 plays an important role in upregulating androgen synthesis in testis. Given the role of MeCP2 in androgenesis, we also evaluated the characterization of the testicular development in the MeCP2^Tg1^ mice, including the testis size, spermatogenesis, and fertility. The results showed that testicular size, spermatogenesis, and fertility of MeCP2^Tg1^ male mice were not significantly different from those of WT mice (Fig. S2).

### MeCP2 regulates androgen synthesis through upregulating LHCGR and downregulating aromatase

**Fig. 2:**
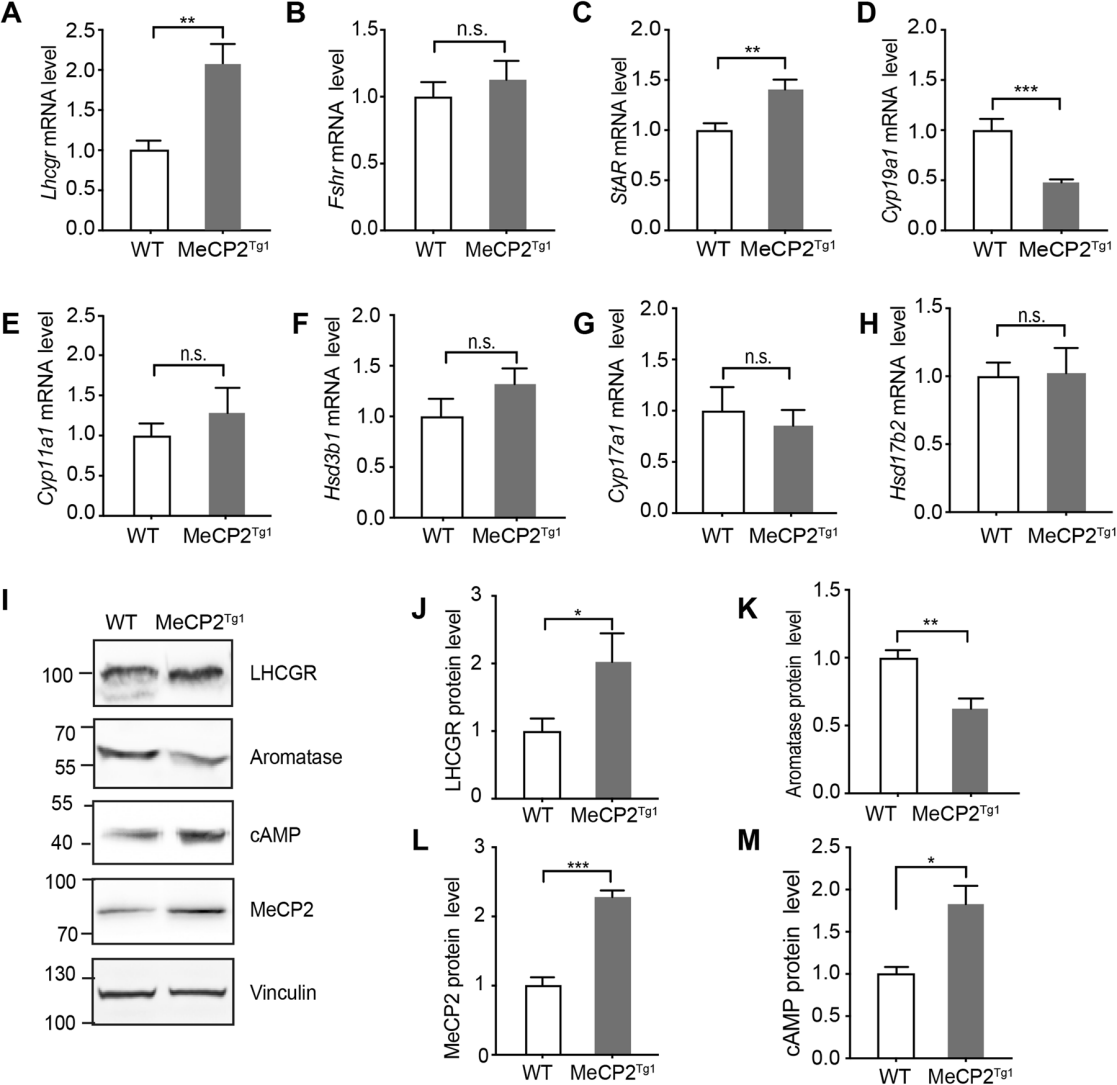
MeCP2 regulates sex hormone metabolism in testis. **A–H** qRT-PCR analysis showing the relative expression of *Lhcgr* (**A**), *Fshr* **(B**), *StAR* (**C**), *Cyp19a1* (**D**), *Cyp11a1* (**E**), *Hsd3b1* (**F**), *Cyp17a1* (**G**), and *Hsd17b2* (**H**) in mouse testis from MeCP2^Tg1^ mice or WT littermates, Mouse GAPDH was used as an internal control. Results were normalized to GAPDH in the same sample. *N* ≥ 5 for each group. **I** Western blot analyses for the expression levels of LHCGR, aromatase, cAMP, and MeCP2 in mouse testis lysates from male MeCP2^Tg1^ mice or WT littermates. Mouse Vinculin was used as the loading control. **J–M** Statistical analyses for the relative expression levels of LHCGR (**J**), aromatase (**K**), MeCP2 (**L**) and cAMP (**M**) in mouse testis as indicated in (**I**). *N* ≥ 5 for each group. For **A**–**H** and **J**–**M**, data are presented as mean ± s.e.m. *p < 0.05; **p < 0.01; ***p < 0.001. n.s. stands for not significant. Statistical analyses were performed using a two-tailed unpaired Student’s *t*-test.

To explore how MeCP2 regulates androgen synthesis in testis, the mRNA levels of LH receptor (*Lhcgr*), FSH receptor (*Fshr*) and several key steroidogenic enzymes were tested in the testis of adult WT or MeCP2^Tg1^ male mice. The results showed that the expression level of *Lhcgr* was significantly upregulated in the testis of MeCP2^Tg1^ mice (Fig. 2A), while the expression of *Fshr* was similar to that in WT mice (Fig. 2B). Also, the expression level of *StAR*, which is activated by *Lhcgr*, was significantly upregulated in the testis of MeCP2^Tg1^ mice (Fig. 2C). Within the key enzymes, the aromatase (*Cyp19a1*), which converts androgen to estrogen, was significantly downregulated in the testis of MeCP2^Tg1^ mice (Fig. 2D). For other enzymes, including P450scc (*Cyp11a1*), 3β-hsd (*Hsd3b1*), P450c17 (*Cyp17a1*), and 17β-hsd (*Hsd17b2*), however, were not significantly affected (Fig. 2E–H). Meanwhile, we also tested the corresponding protein expression levels of LHCGR and aromatase, and found that there were significant upregulated LHCGR and downregulated aromatase in the testis of MeCP2^Tg1^ mice (Fig. 2I–L) and MeCP2-transfected TM3 cells (Fig. S3). The expression of second messengers cAMP was also increased in the testis of MeCP2^Tg1^ mice (Figs. 2I, 2M). Therefore, we deduced that MeCP2 could not only increase androgen synthesis by upregulating LHCGR but also induce androgen accumulation through downregulating aromatase.

### MeCP2 upregulates *Lhcgr* as either a CpG islands binding protein or a G-quadruplex binding protein

**Fig. 3:**
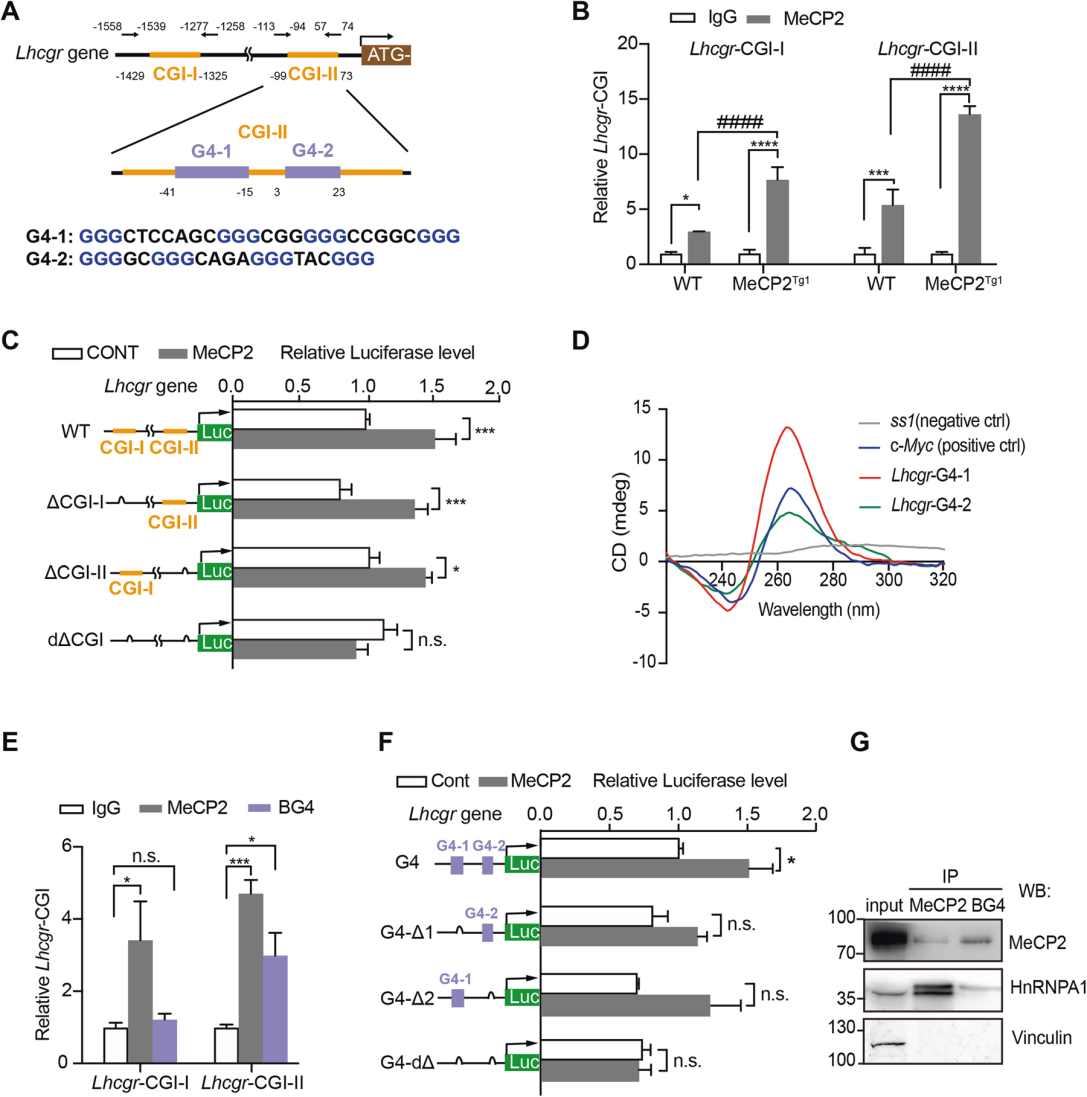
MeCP2 upregulates Lhcgr as either a CpG islands binding protein or a G-quadruplex binding protein. **A** Diagram of the *Lhcgr* gene promoter. Two CpG islands (CGIs, yellow) were located at −1429 bp ~ −1325 bp (CGI-I) and −99 bp ~ 73 bp (CGI-II), respectively; Two putative G-quadruplex sequences (G4, purple) were located at the CGI-II region from −41 bp to −15 bp (G4-1) and 3 bp to 23 bp (G4-2), respectively. The sequences of the two G4s are shown below. **B** ChIP-qPCR analysis showing the relative enrichment of CGI-I and CGI-II regions in chromatin from the testis of MeCP2^Tg1^ and WT mice using the MeCP2 antibodies or control IgG (*N* = 3 for each group). **C** Luciferase activities of the empty vector- (CONT) or MeCP2- transfected TM3 cells co-expressing the indicated plasmids (shown in the left margin). *N* ≥ 3 for each group. **D** Circular dichroism spectra of the *Lhcgr*-G4-1, *Lhcgr*-G4-2, c-*Myc* (positive control), *ss1* (negative control) in 100 mM K^+^ solution. **E** ChIP-qPCR analysis showing the relative enrichment of G-quadruplexes (G4) regions in chromatin from WT mice testis using the MeCP2 antibodies, G-quadruplex antibodies, or IgG. *N* = 3 for each group. **F** Immunoprecipitation assay of BG4-interacting proteins and MeCP2-interacting proteins from TM3 cells transfected with MeCP2 using a BG4 antibody and MeCP2 antibody. The interaction was confirmed by western blot with anti-MeCP2, anti-hnRNPA1, and anti-Vinculin. HnRNPA1, positive control, Vinculin, negative control. **G** Luciferase activities of the empty vector- (CONT) or MeCP2-transfected TM3 cells co-expressing the indicated plasmids (shown in the left margin). *N* ≥ 3 for each group. For **B**, **C** and **E**, **F**, data are presented as mean ± s.e.m. *p < 0.05; ***p < 0.001; ****p < 0.0001, ^####^*p* < 0.0001 and n.s. represents not significant. Statistical analyses were performed with One-way ANOVA.

To evaluate the mechanism of MeCP2 regulating *Lhcgr*, we scanned and analyzed the potential regulatory elements on the promoter of *Lhcgr*. Two CpG islands (CGIs) were identified, which are located at −1,429 bp ~ − 1,325 bp (CGI-I) and − 99 bp ~ 73 bp (CGI-II) of *Lhcgr*’s promoter (Fig. 3A). To explore whether MeCP2 can interact with *Lhcgr* promoter, ChIP-qPCR with MeCP2 antibody was performed in mouse testis. Enriched MeCP2 was detected at both CGI-I and CGI-II sequences in WT mouse testis, and the interactions between MeCP2 and chromatin at these sites were significantly increased in the testis of the MeCP2^Tg1^ mouse (Fig. 3B). The results indicated that MeCP2 could bind to either CGI-I or CGI-II sequences in the *Lhcgr* promoter. Then, we tested the activities of different regions in the *Lhcgr* promoter by luciferase assays to clarify how MeCP2 mediates the activation of *Lhcgr*. Four luciferase reporters were constructed, including the intact CGIs of *Lhcgr* (*Lhcgr*-WT) and three CGI deletion mutants of *Lhcgr* ΔCGI-I (deletion of CGI-I), *Lhcgr* ΔCGI-II (deletion of CGI-II), and *Lhcgr*-dΔCGI (deletion of both CGI-I and CGI-II) (Fig. 3C). The luciferase activities of TM3 cells transfected with *Lhcgr*-WT, *Lhcgr*-ΔCGI-I, or *Lhcgr*-ΔCGI-II were significantly upregulated when co-transfected with MeCP2, while that of *Lhcgr*-dΔCGI co-transfection showed no significant difference under the same condition (Fig. 3C). The results indicated that MeCP2 could regulate *Lhcgr* through both of the two CGIs.

Interestingly, two putative G-quadruplex sequences (PQSs) were found in the CGI-II of the *Lhcgr* gene by QGRS Mapper (https://bioinformatics.ramapo.edu/QGRS/index.php) (Fig. 3A). The two PQSs (*Lhcgr*-G4-1 and *Lhcgr*-G4-2) sequences are relatively conserved in different species, especially in guanine repeating sequences (Fig. S4A), suggesting that these PQSs play important roles in the regulation of *Lhcgr*. PQSs play essential roles in many biological processes, including transcription [18], translation [19], replication, and chromosome stability [20]. Then, we used an optimal fluorescent probe (2-Di-1-ASP) for G-quadruplex nucleic acids to verify the formation of G-quadruplexes in the *Lhcgr* gene in vitro. After the addition of probes, synthetic oligonucleotides of *Lhcgr*-G4-1 and *Lhcgr*-G4-2 in 100 mM KCl fluoresced upon illumination with UV light, while the controls (calf thymus DNA and vehicle) were not (Fig. S4B). Besides, the circular dichroism (CD) spectra of *Lhcgr*-G4-1, *Lhcgr*-G4-2, and *c-Myc* (positive control) in 100 mM KCl showed the representative spectrum of G-quadruplexes, with the maximum absorbance at 265 nm and the minimum at 240 nm, respectively (Fig. 3D). Also, the decreased DNA mobility in gel shift assays suggested the conformational change of *Lhcgr*-G4-1 and *Lhcgr*-G4-2 oligonucleotides resulted from the formation of G-quadruplexes in 100 mM KCl (Fig. S4C). Furthermore, to assess the formation of G-quadruplexes in vivo, we performed ChIP-qPCR with G-quadruplex antibody (BG4) in WT mouse testis. Enrichment of BG4 was detected at the sequences containing PQSs, but not at the other CGI sequences without PGQ in the *Lhcgr* gene (Fig. 3E). These results proved that *Lhcgr* CGI-II sequences contain G-quadruplex structures.

Next, to explore whether the G-quadruplexes in CGI-II are involved in MeCP2 upregulating *Lhcgr* expression, we generated the other four luciferase reporters containing the intact G-quadruplex (*Lhcgr*-CGI-II) and three G-quadruplex deletion mutants, including *Lhcgr*-ΔG4-I (deletion of G4-I), *Lhcgr*-ΔG4-II (deletion of G4-II), and *Lhcgr*-dΔG4 (deletion of both G4-I and G4-II), respectively (Fig. 3F). The luciferase activity of *Lhcgr* CGI-II-transfected TM3 cells was significantly upregulated when co-transfected with MeCP2, while the cells transfected with the three mutants showed no significant difference compared to those co-transfected with MeCP2 (Fig. 3F). Also, we found that both MeCP2 and HnRNPA1 (a known G-quadruplex binding protein) could interact with BG4, while no detectable interaction between BG4 and Vinculin (a negative control protein) (Fig. 3G), suggesting that MeCP2 is indeed a G-quadruplex binding protein. These results implicated that MeCP2 could regulate *Lhcgr* through either CGIs or the G-quadruplex structures.

### G-quadruplex ligands treatment increases the binding of MeCP2 to G-quadruplex and promotes *Lhcgr* transcription by stabilizing the G-quadruplex structure

**Fig. 4:**
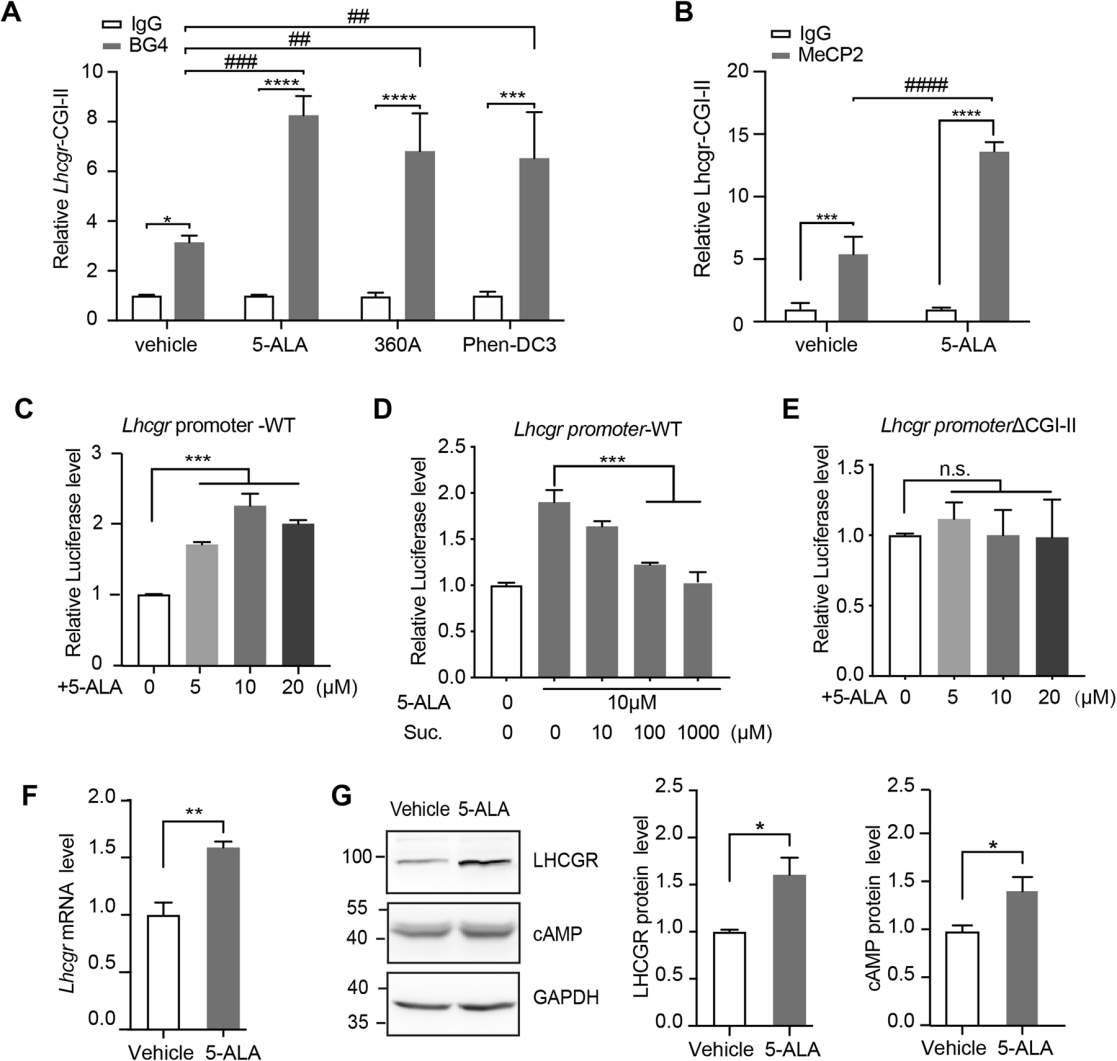
G-quadruplex ligands treatment increases the binding of MeCP2 to G-quadruplex and promotes Lhcgr transcription. **A** The relative enrichment of G-quadruplexes (G4) in *Lhcgr* CGI-II from TM3 cells treated with vehicle or 10 μM 5-ALA, 5 μM 360 A iodide, or 10 μM Phen-DC3 by ChIP-qPCR analysis. The G-quadruplex antibody (BG4) or IgG were used, respectively. *N* = 3 for each group. **B** The relative enrichment of MeCP2 in *Lhcgr* CGI-II from TM3 cells treated with vehicle or 10 μM 5-ALA by ChIP-qPCR analysis. The MeCP2 antibody or IgG was used, respectively. *N* = 3 for each group. **C**, **D** Luciferase activities of the pGL3-*Lhcgr*-WT-transfected TM3 cells with gradient concentrations (5, 10, 20 μM) of 5-ALA treatments (**C**) or co-administered with 10 μM 5-ALA and succinylacetone (Suc.) at 10, 100 or 1000 μM (**D**) for 48 h. The activities were normalized to vehicle-treated cells activities. *N* = 3 for each group. **E** Luciferase activity in TM3 cells transfected with pGL3-*Lhcgr*ΔCGI-II treated with 5-ALA (5, 10, 20 μM) for 48 h. Luciferase activity is shown relative to that in vehicle-treated cells. *N* = 3 for each group. **F** The mRNA level of *Lhcgr* was performed on TM3 cells treated with 10 μM 5-ALA or vehicle, and mouse GAPDH was used as an internal control. *N* = 4 for each group. **G** Western blot analyses showed increased expression of LHCGR and cAMP in TM3 cells with 10 μM 5-ALA treatment than with vehicle (ddH_2_O). Mouse GAPDH was used as the internal control. Quantification (right) of western blot results (*N* ≥ 3) normalized to GAPDH values. For **A**–**G**, data are presented as mean ± s.e.m. *p < 0.05; ***p < 0.001; ****p < 0.0001, ^##^*p* < 0.01, ^###^*p* < 0.001, ^####^*p* < 0.0001 and n.s. represents not significant. Statistical analyses were performed with One-way ANOVA (**A**–**E**, **G**) or two-tailed unpaired Student’s *t*-test (**F**).

G-quadruplex ligands can stabilize or destabilize the conformation of G-quadruplex helices and function as transcriptional regulators. Therefore, we wondered what the effects of the G-quadruplex ligands on the function of G-quadruplex on *Lhcgr* were. Three G-quadruplex ligands [5-aminolevulinic acid (5-ALA), 360 A iodide, and Phen-DC3 Trifluoromethanesulfonate (Phen-DC3 Triflate)] were tested, and we found that the interactions between BG4 and the tested G-quadruplex of *Lhcgr* were significantly increased with the treatment of 5-ALA, 360 A iodide, or Phen-DC3 Triflate in TM3 cells (Fig. 4A). The results indicated that the treatment with G-quadruplex ligands stabilized *Lhcgr* G-quadruplex. As expected, there was no interaction between BG4 and *Lhcgr* CGI-I, and the G-quadruplex ligands showed no effect on CGI-I (Fig. S5). Moreover, the treatment of G-quadruplex ligands increased the binding of MeCP2 to G-quadruplex (Fig. 4B) and promoted *Lhcgr* transcription. Specifically, the treatment with gradient concentrations of 5-ALA significantly activated the luciferase activity of *Lhcgr*-WT-transfected TM3 cells (Fig. 4C), and the increased activity could be reversed by the 5-ALA dehydratase inhibitor succinylacetone (Fig. 4D). However, the activity of *Lhcgr* ΔCGI-II-transfected cells showed no significant difference (Fig. 4E). Furthermore, 5-ALA upregulated the expression of LHCGR and cAMP in TM3 cells (Fig. 4F, G). These results suggested that G-quadruplex ligands treatment activates *Lhcgr* gene expression through increasing the binding of MeCP2 to G-quadruplex.

### MeCP2 binds to *Lhcgr* G-quadruplex structures and recruits CREB1 to promote *Lhcgr* transcription

**Fig. 5:**
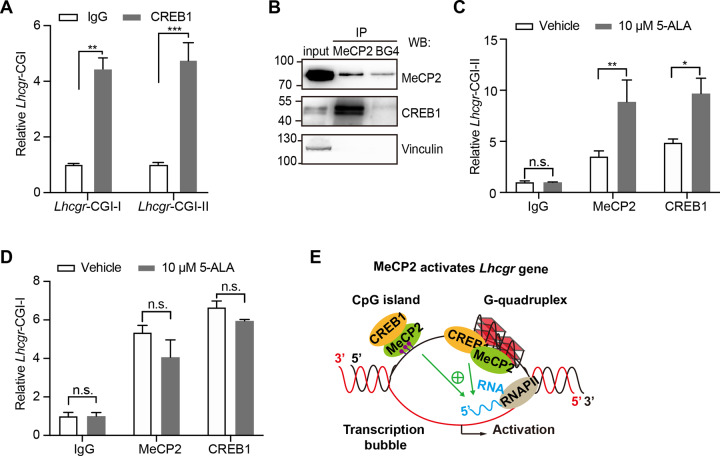
MeCP2/CREB1 complex binds to *Lhcgr* G-quadruplex structures and CGIs. **A** ChIP-qPCR analysis is showing the relative enrichment of CGI-I and CGI-II regions in chromatin from the testis of WT mice using the CREB1 antibodies or control IgG. *N* = 3 for each group. **B** Immunoprecipitation assay of BG4-interacting proteins and MeCP2-interacting proteins from TM3 cells transfected with MeCP2 using a BG4 antibody and MeCP2 antibody. The interaction was confirmed by western blot with anti-MeCP2, anti-CREB1, and anti-Vinculin. Vinculin was used as a negative control. **C**, **D** The relative enrichment of *Lhcgr*-CGI-II (**C**) or *Lhcgr*-CGI-I (**D**) in *Lhcgr* with or without 10 μM 5-ALA treatment by ChIP-qPCR analysis in TM3 cells. The MeCP2, CREB1 antibodies, or IgG were used, respectively. *N* = 3 for each group. **E** Schematic illustration of the mechanism of MeCP2 regulation on *Lhcgr*. For **A**, **C**, and **D**, data are presented as mean ± s.e.m. *p < 0.05; **p < 0.01; ***p < 0.001, n.s. represents not significant. Statistical analyses were performed with One-way ANOVA.

A previous study showed that MeCP2 recruits the transcriptional activator CREB1 on the promoter of the target gene to promote transcription [21]. To explore whether CREB1 is involved in MeCP2-activated *Lhcgr* transcription, ChIP analysis with primers targeting to the two CGIs of *Lhcgr* was performed in the WT mouse testis. Substantial CREB1 enrichment was detected at the two CGIs of *Lhcgr* (Fig. 5A). Also, we found that both of MeCP2 and BG4 could interact with CREB1 (Fig. 5B). Besides, treatment with 5-ALA increased the MeCP2/CREB1 interaction with *Lhcgr* CGI-II at which the promoter contains G-quadruplexes (Fig. 5C), while *Lhcgr* CGI-I was not (Fig. 5D). These results suggested that MeCP2/CREB1 complex binds to *Lhcgr* G-quadruplex structures and CGI, enhancing the expression of *Lhcgr* (Fig. 5E).

### MeCP2 downregulates *Cyp19a1* via inhibition of *Rorα*

**Fig. 6:**
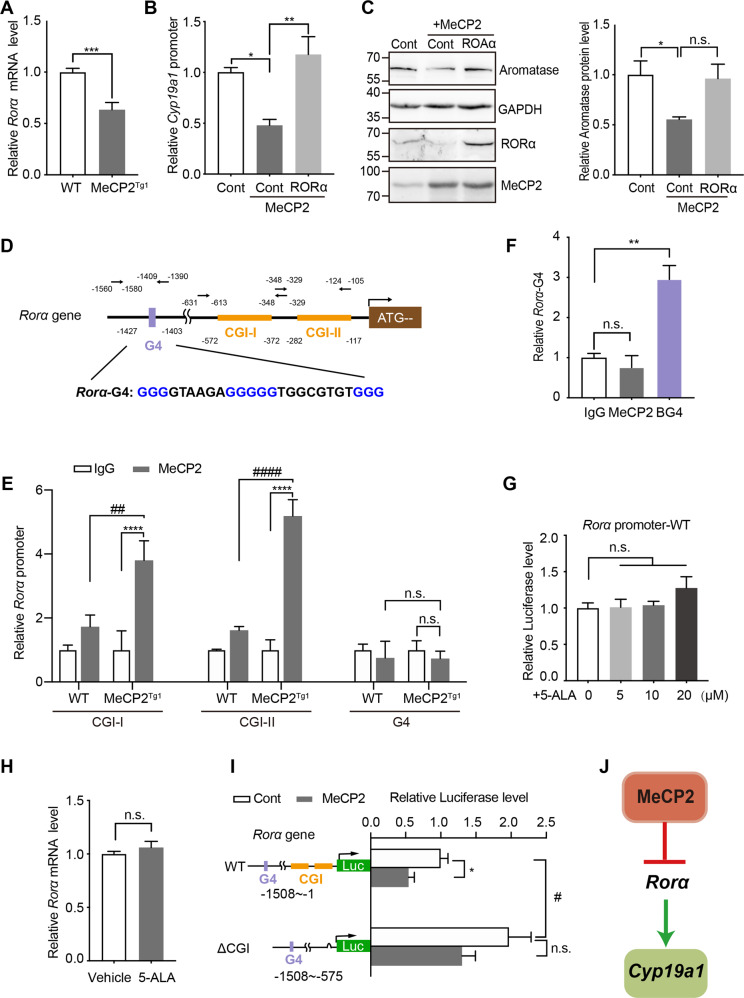
MeCP2 regulates *Cyp19a1* via RORα. **A** qRT-PCR analysis showing the relative expression of *Rorα* in mouse testis from MeCP2^Tg1^ mice or WT littermates, Mouse *GAPDH* was used as an internal control. Results were normalized to *GAPDH* in the same sample. *N* = 6 for each group. **B** Luciferase activities of TM3 cells co-transfected with luciferase reporter plasmids of *Cyp19a1* promoter plus other indicated plasmids: control, MeCP2 or MeCP2 plus RORα. N = 3 for each group. **C** Western blot analyses showed that overexpression of RORα together with MeCP2 could reverse the effect of MeCP2 on downregulating aromatase in TM3 cells. TM3 cells lysates were subjected to Western blot analysis for aromatase, RORα, and MeCP2. GAPDH was used as a loading control. Representative blots are shown on the left panel, and statistical analyses were presented on the on the right panel. **D** Diagram of the *Rorα* promoter. Two CpG islands (CGIs, yellow) were located at −572 bp ~ −372 bp (CGI-I) and −282 bp ~ −117 bp (CGI-II), respectively; And putative G-quadruplex sequences (G4, purple) was located at −1427 bp ~ −1403 bp, respectively. The G4 sequence is shown below. **E** ChIP-qPCR analysis showing the relative enrichment of the CGI-I, CGI-II, and G4 regions in chromatin from the testis of MeCP2^Tg1^ and WT mice using the MeCP2 antibody, BG4 antibody, or control IgG, respectively. *N* = 3 for each group. **F** Analysis of *Rorα* G4 regions in chromatin from the testis of MeCP2^Tg1^ and WT mice by ChIP-qPCR with the MeCP2 antibody, BG4 antibody, or control IgG. *N* = 3 for each group. **G** Luciferase activities of the pGL3-*Rorα*-promoter-transfected TM3 cells with gradient concentrations (5, 10, 20 μM) of 5-ALA treatments. The activities were normalized to vehicle-treated cells activities. *N* = 3 for each group. **H** qRT-PCR analyses showed no increased expression of *Lhcgr* in TM3 cells with 10 μM 5-ALA treatment than with vehicle (ddH_2_O). Mouse *GAPDH* was used as the internal control. *N* = 3 for each group. **I** Luciferase activities of the empty vector (Cont) or MeCP2-transfected TM3 cells co-expressing the different truncated *Rorα* promoter plasmids (shown in the left margin). *N* ≥ 3 for each group. **J** Schematic diagram of MeCP2 regulation on *Cyp19a1* via RORα. For **A**–**C**, **F**–**I**, data are presented as mean ± s.e.m. *p < 0.05; **p and ^##^*p* < 0.01; ***p < 0.001; ^####^*p* < 0.0001, n.s. represents not significant. Statistical analyses were performed using two-tailed unpaired Student’s *t*-test (**A**, **H**) or One-way ANOVA (**B**, **C**, **F**, **G**, and **I**).

Since the expression of the Cyp19a1 gene was significantly downregulated in the testis of MeCP2^Tg1^ mice (Fig. 2D), we wondered how MeCP2 regulates *Cyp19a1*. ChIP-qPCR assays showed that MeCP2 could not bind to the promoter of *Cyp19a1* directly (Fig. S6). A previous study reported that RORα, a member of the nuclear receptor superfamily of transcription factors, could directly target *Cyp19a1* [22], so we investigated whether RORα was involved in the MeCP2 downregulation of *Cyp19al*. We found significantly decreased *Rorα* mRNA in MeCP2^Tg1^ testis than that in WT mouse testis (Fig. 6A). To determine whether MeCP2 regulates *Cyp19a1* via RORα, we cloned *Cyp19a1* genomic sequences containing RORα response elements (ROREs) into a pGL3-control luciferase reporter vector. The luciferase assays showed that the activity of pGL3-*Cyp19a1*-transfected TM3 cells was significantly reduced when co-transfected with MeCP2 (Fig. 6B). However, the luciferase activity was rescued when RORα was co-transfected with MeCP2 (Fig. 6B), suggesting that RORα was able to reverse the MeCP2-induced downregulation of luciferase activity. In addition, the protein expression of aromatase was also reversed when RORα was co-transfected with MeCP2 in TM3 cells. (Fig. 6C). These results revealed that MeCP2 regulates *Cyp19a1* through RORα.

Then, we investigated how MeCP2 regulates *Rorα*. There are two CGIs (CGI-I and CGI-II) and a sequence potentially forming a G-quadruplex structure in *Rorα*’s promoter (Fig. 6D). To determine the location of MeCP2-interacting targets in *Rorα*’s promoter, we performed ChIP-qPCR with MeCP2 antibody and luciferase reporter assays. Increased MeCP2 were detected at either *Rorα*’s CGI-I or CGI-II in MeCP2^Tg1^ mice testis (Fig. 6E). However, MeCP2 didn’t interact with chromatin at the sequence potentially forming G-quadruplex (Fig. 6F) in *Rorα*’s promoter. Besides, 5-ALA didn’t affect the *Rorα* expression (Fig. 6G-H). In addition, MeCP2 is known to bind to methylated-CpG of DNA and acts as a transcriptional repressor association with SIN3A and histone deacetylases. We found that SIN3A can interact with the sequences containing CGIs of the *Rorα* gene (Fig. S7A), while CREB1 doesn’t bind to the G4 or CGI in the promoter of *Rorα* (Fig. S7B).

Also, to explore whether MeCP2 downregulates *Rorα* expression through binding to its CpG island, we constructed luciferase reporters of *Rorα*-WT (with a 1387 bp fragment of *Lhcgr*’s promoter covering this CpG island) and *Rorα*-ΔCGI (deleting the site of CpG island in the *Rorα*’s promoter). Then the two constructs were co-transfected into TM3 cells with either MeCP2 or the vector. We found that MeCP2 significantly downregulated the relative luciferase level of *Rorα*-WT but showed no effect on *Rorα*-ΔCGI (Fig. 6I). These results suggested that MeCP2 could reduce the expression of *Rorα* by interacting with *Rorα*’s CGI island. Therefore, we confirmed that Rorα is inhibited by MeCP2 through the CGI of *Rorα*, which contributes to the decrease of *Cyp19a1* expression in MeCP2^Tg1^ mice testis (Fig. 6J).

## Discussion

**Fig. 7:**
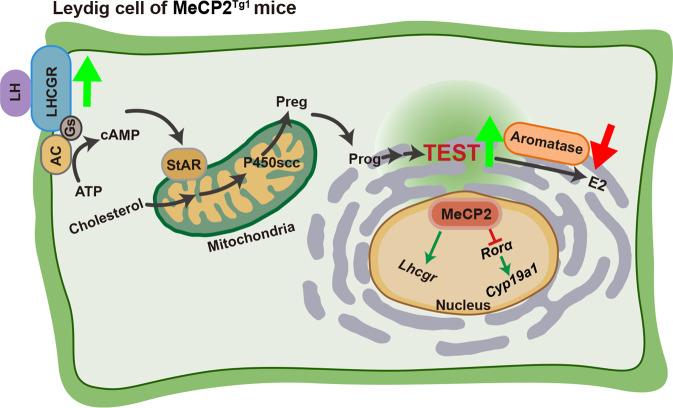
Schematic illustration for MeCP2 regulating androgen via LHCGR and aromatase. The illustration shows MeCP2 regulates its target genes (*Lhcgr*, *Rorα*) in the nucleus of the Leydig cell. The bold arrows (green or red) indicate that MeCP2 is involved in the testicular steroidogenesis by upregulating LHCGR and downregulating aromatase. TEST, testosterone.

In this study, we found that androgen was elevated in the MDS mouse model (MeCP2^Tg1^ mice), similar to the phenotype of MDS patients. We further showed that MeCP2 was relatively highly expressed in Leydig cells which were located at the connective tissue surrounding the sperm-producing seminiferous tubules of the testes and were involved in the production and secretion of testosterone. These findings suggest that MeCP2 plays an important role in the process of androgen synthesis. Also, we discovered that MeCP2 promoted androgen synthesis by upregulating *Lhcgr* and suppressing the conversion of androgen to estrogen by downregulating *Cyp19a1* (Fig. 7). Our results support a novel non-CNS function of MeCP2 in the process of sex hormone synthesis.

In addition to profound neurological function, MeCP2 is also involved in the pathologies of many tissues and cells. Previous researches have reported that MeCP2 protein is highly expressed in the lung, spleen, kidney, and heart, and as a transcriptional regulator, it has attracted more and more attention in various tissues and cells. For example, overexpression of MeCP2 causes immune dysfunction by suppressing IFN-γ production from T_H_1 cells [6]. And a study has found heart and bone abnormalities caused by local overexpression of transgenic MeCP2 embryos [23]. Our study is the first to report MeCP2 is expressed in the Leydig cells of the testis and explore its functions in androgen biogenesis.

As a transcriptional repressor or activator, MeCP2 occupies a large proportion of the genome promiscuously [24, 25], including methylated DNA such as cytosines in CpG dinucleotides (mCG), methylated CA (mCA) sites, and non-methylated DNA. However, few evidence suggests that MeCP2 can regulate gene expression by targeting single *cis* elements or specific motifs on genes. Here, we report a novel role for MeCP2 as a reader of G-quadruplexes when it regulates *Lhcgr*. G-quadruplexes are dynamic structures folded in G-rich DNA and RNA regions. One of the widely used consensus putative G-quadruplex sequences (PQS) is G ≥ 3 N1-7 G ≥ 3 N1-7 G ≥ 3 N1-7 G ≥ 3, wherein G-tracts are connected by loops of varying length and nucleotide (N) composition [26, 27]. G-quadruplex forming sequences are widespread throughout the genome, especially enrichment in repetitive sequences such as telomere and also in promoter regions close to the transcription start site (TSS) [28]. G-quadruplexes are often formed in the domains of negatively supercoiled DNA, and they could be stabilized and regulated by protein interaction [29]. G-quadruplexes in promoter regions can influence transcription efficiently [30]. Among G-quadruplex-binding proteins in gene promoters, many of them are involved in transcriptional regulation such as PARP1(Poly (ADP-Ribose) Polymerase 1) [31], as well as in chromatin remodeling and DNA repairs such as CNBP (cellular nucleic acid-binding protein) [32], MAZ (myc-associated zinc-finger) [33] and hnRNPA1(Heterogeneous Nuclear Ribonucleoprotein A1) [34]. Here, we identify MeCP2 as a novel G-quadruplex-binding protein, binding to the G-quadruplex of *Lhcgr* in mouse testis and regulating its expression by recruiting the activator CREB1. In addition, G-quadruplexes have important roles in neurological diseases, including G-quadruplex-forming repeat expansions in the *C9orf72* gene in frontotemporal dementia and amyotrophic lateral sclerosis [35, 36] and loss of the G-quadruplex binding protein FMRP in the intellectual disability fragile X syndrome [37]. Whether G-quadruplexes are targets for MeCP2 in the brain is unknown. We roughly screened potential genes which might form G-quadruplex structures regulated by MeCP2 and compared the genes regulated by MeCP2 with the genes that contained consensus putative G-quadruplex sequences [38], about 40% (1034/2558) of MeCP2-regulated genes overlapped with the genes that contained PQSs (Fig. S8). Furthermore, the human genes contained PQSs in the promoter (−2000~0 bp) are highly correlated with NEUROGENESIS analyzed with GSEA. Further study remains needed to better understand the function of MeCP2 as a G-quadruplex reader in brain development and neurological diseases.

Previous clinical studies [10] and current studies all showed that MeCP2 duplication could cause elevated testosterone. Studies have shown that fetal testosterone is the organizing mechanism for sexual dimorphism in the human brain [39–41]. Testosterone has also been shown to be closely related to many autistic behaviors, including social and emotional behavior, and the level of testosterone in male autistic patients is positively correlated with the severity of the disease [42, 43]. Moreover, some studies have even shown differences in the expression of genes involved in sex hormone biosynthesis between autistic individuals and controls [44, 45]. In the present study, we showed that the testosterone level elevated and sex hormone biosynthesis was abnormal in the male MDS mice. These findings suggest that sex hormones may be relevant to the function of the brain, contributing to male individuals’ increased susceptibility to autistic features. As a result, sex hormones have the potential to be a new treatment for neurodevelopmental disorders such as autism in the future.

In summary, we found that in addition to neurological dysfunction, duplication of the *MECP2* gene led to disturbances in sex hormone synthesis due to the upregulated androgen synthesis pathway and reduced androgen conversion to estrogen in testis. Abnormal expression of genes related to hormone synthesis and elevated androgen levels in MDS and autism may contribute to autism symptoms and central nervous system disorders. In addition, we investigated a novel mechanism by which MeCP2 acts as a G-quadruplex reader to regulate gene expression in the testis, which may be a new therapeutic target for MDS.

## Materials and methods

### Animals

FVB-Tg (*MECP2*)1Hzo/J (MeCP2^Tg1^) mice were maintained in the animal facility at the Institute of Developmental Biology & Molecular Medicine, Fudan University. The genotyping of MeCP2^Tg1^ mice was determined by PCR following the protocols from the Jackson Laboratory.

### Cell culture and drugs exposure

TM3 cell line was grown in DMEM/F-12 supplemented with 5% horse serum, 2.5% fetal bovine serum, and penicillin/streptomycin (100 units per 100 μg ml^–1^) in a 5% CO_2_ incubator at 37 °C. In addition, 10 ng/mL human luteinizing hormone (hCG/LH) (GLPBIO, Cat# GC39581) was added for the stimulation of testosterone synthesis. Transfection was performed using FuGENE (Roche, Cat#14738300) transfection reagent according to the manufacturer’s protocol. When the volume of adherent cells occupied 70% of the dish, the cells were treated with 10 μM of 5-ALA (MCE, Cat. No. HY-N0305), 360 A iodide (MCE, Cat. No. HY-15595A), Phen-DC3 Triflate (MCE, Cat# HY-15594A), Succinyl phosphonate (MCE, Cat# HY-12688) or vehicle (distilled water) for 48 h. Each treatment was replicated at least three times.

### qRT-PCR and western blot

Total RNA from hypothalamus, pituitary, testis, or TM3 cells was extracted with TRIzol (Invitrogen) and reverse transcribed (Toyobo, Cat# FSQ-301) for qRT-PCR. Real-time PCR was performed using Realtime PCR Master Mix (Toyobo, Cat# QPK-101). Relative expression of each target gene was calculated by comparison to the expression of mouse *GAPDH* (Primers used, see Table S1). For Western blot, cells were homogenized in ice-cold RIPA buffer and centrifuged at 12,000 rpm. The supernatant was resolved by 10% SDS-PAGE, electro-transferred to a PVDF membrane, and probed with the following antibodies: rabbit anti-MeCP2 (Cell Signaling Technology; Cat# 3456; 1:1 000), rabbit anti-aromatase (Abcam; Cat# ab18995; 1:500), rabbit anti-LHCGR (Proteintech; Cat# 19968-1-AP; 1:1 000), rabbit anti-Vinculin (Abcam; Cat# ab129002; 1:5 000), bovine anti-goat IgG-HRP (Santa Cruz Biotechnology; Cat# sc2352; 1:4,000), and goat anti-rabbit IgG-HRP (Beyotime; Cat# A0208; 1:1 000) antibodies. Blotting images were acquired with a Tanon-5200 imaging system. Densitometric quantification of the target protein was determined by Image J and compared with the internal control to determine the relative expression value. The internal control was Vinculin. The representative blots were selected from at least three repeated experiments.

### H&E staining

Fresh testicular tissue samples from both WT and MeCP2^Tg1^ male mice were fixed in Bouin’s solution (Polysciences) and embedded in paraffin. Sections were cut at a 5-μm thickness on a Leica RM2165 Microtome and stained with Hematoxylin-Eosin / HE Staining Kit (Solarbio, G1120).

### Circular dichroism (CD) spectra

Oligonucleotides for CD spectra were prepared in Na^+^ solution or K^+^ solution (10 or 100 mM). Annealing was performed by heating to 95 °C for 5 min and cooling down slowly to room temperature for 4 h. CD spectra were measured in 0.5-nm steps from 340 to 220 nm using a Chirascan spectrometer and 1 mm quartz cuvettes.

### Gel electrophoresis

DNA samples for gel electrophoresis were prepared similarly to CD spectra samples. Annealing was performed by heating to 95 °C for 5 min and cooling slowly to room temperature. Each sample was analyzed by native gel (8%) in 0.5× TBE buffer at room temperature. Single-stranded DNA was stained with Gelred (50513, Lonza). Gels were then imaged by Tanon 2500B.

### ELISA

TM3 cells were seeded in 12-well plates (~1 × 10^5^ cells/well) and treated with Luteinizing hormone (human). Mouse serum (20 µL) and TM3 cell supernatant (100 µL) were collected and diluted to 5% and 50%, respectively. Then total testosterone was measured in the media by Testosterone ELISA Kit (Abnova, Cat# KA0309). Serum luteinizing hormone (LH) was measured by Abnova™ LH (Rodent) ELISA Kit (Abnova™, Cat# KA2332). Serum follicle-stimulating hormone (FSH) was detected by Elabscience^®^ Mouse FSH (Follicle-Stimulating Hormone) ELISA Kit (Cat# E-EL-M0511c). Serum estradiol was detected by Elabscience^®^ Human/Monkey/Mouse E2 (Estradiol) ELISA Kit (Cat# E-EL-0150c). Each experiment was conducted in triplicate, with three wells per treatment in each experiment.

### Optimal fluorescent probe for detecting G-quadruplex

DNA samples for Optimal fluorescent probe detecting were prepared similarly to CD spectra samples. Vehicle, G4-1(10 μM), G4-2(10 μM), or dsDNA (ctDNA, 250 μM bp) mixed with 5 μM 2-Di-1-ASP (MCE, Cat# HY-135009) illuminate with UV light (*λ* = 312 nm), fluorescence maps were then imaged by Tanon 2500B. The sequences of probes were listed in Table S2.

### Chromatin immunoprecipitation (ChIP) assay

The testis of WT mice or MeCP2^Tg1^ mice was isolated and cut into small cubes (1–3 mm^3^) for cross-linking with 1% formaldehyde at 37°C for 25 min. Stopped the reaction with 0.2 M Glycine for 10 min at room temperature (RT), and then sedimented, washed, homogenated, and lysed with SDS lysis buffer at 4 °C for 10 min. The lysates were sonicated to reduce DNA lengths in the ice and were subjected to the chromatin immunoprecipitation assay using previously described protocols. Cross-linked protein-DNA complexes were immunoprecipitated by incubating with rabbit anti-MeCP2 antibody (Abcam, Cat# ab2828), rabbit anti-CREB antibody (Cell Signaling, Cat# 9197 S), anti-DNA G-quadruplex structures antibody, clone BG4 (Sigma-Aldrich, Cat# MABE917), anti-SIN3A antibody (Cell Signaling, Cat# 7691) and rabbit IgG (negative control) antibodies overnight and then with Protein A + G Agarose beads at 4 °C for 2 h. DNA was thereafter purified from the mixture. The DNA fragments were quantitatively amplified by real-time PCR. Primers used see Table S3.

### Immunoprecipitation and immunoblotting

Immunoprecipitation and immunoblotting analysis were performed as previously described. Briefly, TM3 cells were lysed with SDS lysis buffer at 4°C for 10 min. For immunoprecipitation, lysates were incubated overnight at 4 °C with anti-MeCP2 antibody (Abcam, Cat# ab2828), rabbit anti-CREB antibody (Cell Signaling, Cat# 9197 S), anti-DNA G-quadruplex structures antibody, clone BG4 (Sigma-Aldrich, Cat# MABE917), and rabbit anti-HnRNPA1(Cell Signaling, Cat# 8443), and then with Protein A + G Agarose beads (Santa Cruz Biotechnology, Cat# sc-2003) at 4°C for 2 h. Subsequently, immunoprecipitants were washed three times, equivalent amounts of protein were detected by Western blot with rabbit anti-MeCP2 antibody (Cell Signaling Technology; Cat# 3456; 1:1000).

### Plasmid construction

Human MECP2-e1 cDNA was purchased from Origene (RC202382). The *Rorα* expression plasmid containing mouse *Rorα* cDNA was amplified by PCR and subcloned into the EcoRI/KpnI sites of pCMS-EGFP plasmid. Seven different regions of mouse Lhcgr-promoter were cloned into XbaI/FseI sites of a pGL3-control plasmid to generate luciferase reporter constructs. *Lhcgr*-WT contains −1558 bp ~ 84 bp of the *Lhcgr* promoter, which covers the predicted two CpG islands (−1429 bp ~ −1325 bp and −99 bp ~ 73 bp) and G-quadruplex (−41 bp ~ −15 bp, 3 bp ~ 23 bp). The double CGI deletion reporter *Lhcgr*-dΔCGI which deleted the two CGIs contains −1429 bp ~ −99 bp of the *Lhcgr* promoter. *Lhcgr*-CGI-II contains −89 bp ~ 88 bp of the *Lhcgr* promoter, which covers the predicted two G-quadruplex-forming sequences (−41 bp ~ −15 bp, 3 bp ~ 23 bp). The single quadruplex deletion reporters *Lhcgr*ΔG4-I and *Lhcgr*ΔG4-II, which deleted one of the G-quadruplex-forming sequences of the *Lhcgr*-CGI-II. The double quadruplex deletion reporter *Lhcgr*-dΔG4, which deleted the two G-quadruplex-forming sequences of the *Lhcgr*-CGI-II. The sequences of different *Lhcgr* promoter truncations are listed in Table S4.

Mouse *Cyp19a1* promoter (−1493 bp ~ −1 bp) and mouse *StAR* promoter (−1442 bp ~ −1 bp) were cloned into KpnI/BglII sites of pGL3 plasmid to generate luciferase reporter constructs. Two different regions of mouse Rorα-promoter were cloned into KpnI/BglII sites of pGL3 plasmid to generate luciferase reporter constructs. *Rorα*-WT contains −1487 bp ~ −1 bp of the *Rorα* promoter, which covers the predicted CpG islands (−551 bp ~ −96 bp) and G-quadruplex (−1407 bp ~ −1382 bp). Primers used, see Table S5.

### Quantification and statistical analysis

All experiments were repeated at least three times, and the statistical significance was evaluated. Data are expressed as mean ± SEM. Statistical differences were calculated by a two-tailed unpaired *t*-test for two datasets and ANOVA followed by Bonferroni post-hoc test for multiple datasets using Prism (GraphPad Inc., La Jolla, CA). *p* < 0.05 was considered statistically significant.

## Supplementary information

Supplementary materials

Figure S1

Figure S2

Figure S3

Figure S4

Figure S5

Figure S6

Figure S7

Figure S8

## Data Availability

The datasets generated during and/or analyzed during the current study are available from the corresponding author on reasonable request.
