# Supplementary material for: MeCP2 duplication causes hyperandrogenism by upregulating LHCGR and downregulating RORα
Source: Cell Death Dis. 2021 Oct 25;12(11):999. doi: 10.1038/s41419-021-04277-4 (PMC8545957; doi:10.1038/s41419-021-04277-4)
Supplement: Supplementary file 1 — Supplementary materials [file 41419_2021_4277_MOESM1_ESM.docx]

**Supplementary materials**

**Supplementary figure legends:**

**Figure S1 Elevated androgen in MeCP2^Tg1^ mice.** Real-time RT-qPCR showing the mRNA level of *Gnrh1* in mouse hypothalamus lysates **(A)**, *Lhb* **(B)** and *Gnrhr* **(C)** in pituitary lysates from MeCP2^Tg1^ mice and WT littermates, mouse *Gapdh* was used as internal control. N ≥ 4 for each group. All data were analyzed by two-tailed unpaired Student’s *t*-test. Data are presented as mean ± s.e.m. n.s. represents not significant.

**Figure S2 General characterization of the testis development of WT and MeCP2^Tg1^ male mice. (A)** Comparison of the testis sizes of WT and MeCP2^Tg1^ male mice. Scale bar, 1mm. **(B)** Comparison of the relative testis weight / body weight between WT (N=17) and MeCP2^Tg1^ (N=16) male mice. **(D)** Hematoxylin and eosin-stained testis sections from WT and MeCP2^Tg1^ mice testis. The spermatogenesis of MeCP2^Tg1^ mice is normal. The development of sperm flagella was investigated in mouse testis through the use of H&E staining. In stage VII–VIII seminiferous tubules, normal round spermatids (arrowheads) and elongated tails (arrows) were observed in the testes from MeCP2^Tg1^ male mice. Scale bar: 100 μm **(C1, C3)**; 20 μm **(C2, C4)**. **(E)** Comparison of the fertility between WT and of MeCP2^Tg1^ mice. Each male mouse was bred with two WT female mice, and the pups were measured. For **(B)** and **(D),** data are presented as mean ± s.e.m. n.s. represents not significant. Statistical analyses were performed using two-tailed unpaired Student’s *t*-test.

**Figure S3 MeCP2 regulates sex hormone metabolism in Leydig cells. (A)** Western blot analyses for the expression levels of LHCGR, Aromatase and MeCP2 in TM3 cells. The cells were transfected with vector (CONT) or MeCP2-expressing plasmid (MeCP2), respectively. Mouse Vinculin was used as the loading control. **(B-D)** Statistical analyses for the relative expression levels of LHCGR, Aromatase and MeCP2 in TM3 cells as indicated in (I). N = 7 for each group. All data were analyzed by two-tailed unpaired Student’s *t*-test. data are presented as mean ± s.e.m. n.s. represents not significant.

**Figure S4 MeCP2 is a G-quadruplex binding protein upregulating LHCGR. (A)** Sequence alignments of LHCGR G4 from different species. Strictly conserved and similar residues are highlighted with a red box and by a red letter, respectively. The sequence alignment figure was generated by Clustal Omega and ESPript 3.0. **(B)** Photographs of vehicle, G4-1(10 μM), G4-2(10 μM), or dsDNA (ct DNA, 250 μM bp) in 10 mM or 100 mM Na^+^/K^+^ mixed with 2-Di-1-ASP (5 μM) upon bottom illumination with UV light (λ = 312 nm). **(C)** The DNA of *Lhcgr*-G4-1 or *Lhcgr*-G4-2 were prepared in Na^+^ solution or K^+^ solution (10 or 100 mM). Each sample was analyzed by native gel.

**Figure S5 G-quadruplex ligands treatment increases the binding of MeCP2 to G-quadruplex and promotes *Lhcgr* transcription.** The relative enrichment of G-quadruplexes (G4) Lhcgr CGI-II from TM3 cells treated with vehicle or 10 μM 5-ALA, 5 μM 360A or 10 μM Phen-DC3 by ChIP-qPCR analysis. The G-quadruplex antibody (BG4) or IgG were used, respectively. N = 3 for each group.

**Figure S6 MeCP2 regulates *Cyp19a1* via RORα.** Analysis of different regions of *Cyp19a1* promoter in chromatin from the testis of MeCP2^Tg1^ and WT mice by ChIP-qPCR with the MeCP2 antibodies or control IgG. n.s. represents not significant by two-tailed unpaired Student’s *t*-test. Data are presented as mean ± s.e.m. N=3 for each sample.

**Figure S7 MeCP2 downregulates Rora associated with SIN3A rather than CREB1. (A)** ChIP-qPCR analysis showing the relative enrichment of SIN3A in *Rora* CGI-I, CGI-II, and G-quadruplex regions in chromatin from TM3 cells using the SIN3A antibodies or control IgG (N = 3 for each group). **(B)** Analysis of *Rorα* CGI-I, CGI-II, and G-quadruplex regions in chromatin from the TM3 cells by ChIP-qPCR with the CREB1 antibody or control IgG. N = 3 for each group.

**Figure S8 Venn diagram showing about 40% MeCP2-regulated genes (1034/2558) overlapped with the genes contained PQSs.**

**Supplementary tables:**

**Table S1 The primers used for qRT-PCR.**

| **Gene name** | **Primer** |
| --- | --- |
| Mouse *Gapdh* | F: 5’- ACAGCAACTCCCACTCTTCCACCT -3’  R: 5’- TTGCTCAGTGTCCTTGCTGGGG -3’ |
| Mouse *Lhcgr* | F: 5’- CGCCCGACTATCTCTCACCTA -3’  R: 5’- GACAGATTGAGGAGGTTGTCAAA -3’ |
| Mouse *Fshr* | F: 5’- CCTTGCTCCTGGTCTCCTTG -3’  R: 5’- CTCGGTCACCTTGCTATCTTG -3’ |
| Mouse *Cyp11a1* | F: 5’- AGGTCCTTCAATGAGATCCCTT -3’  R: 5’- TCCCTGTAAATGGGGCCATAC -3’ |
| Mouse *Cyp19a1* | F: 5’- AACCCCATGCAGTATAATGTCAC -3’  R: 5’- AGGACCTGGTATTGAAGACGAG -3’ |
| Mouse *Rorα* | F: 5’- GTGGAGACAAATCGTCAGGAAT -3’  R: 5’- TGGTCCGATCAATCAAACAGTTC -3’ |
| Mouse *Hsd3b1* | F: 5’- AGCTCTGGACAAAGTATTCCGA -3’  R: 5’- GCCTCCAATAGGTTCTGGGT -3’ |
| Mouse *Cyp17a1* | F: 5’- GCCCAAGTCAAAGACACCTAAT -3’  R: 5’- GTACCCAGGCGAAGAGAATAGA -3’ |
| Mouse *Hsd17b2* | F: 5’- ACCTTGTTCCTCTTATCCGTGG -3’  R: 5’- ACCGAAACCGGAATCAGCAC -3’ |

**Table S2** **Optimal fluorescent probe for G-quadruplex nucleic acids.**

| **G-quadruplex** | **The sequence of probe** |
| --- | --- |
| c-*Myc* G-quadruplex | 5’- TGAGGGTGGGTAGGGTGGGTAA -3’ |
| *ss1* (negative control) | 5’- CACTAAACCTAACACTAACCAT -3’; |
| *Lhcgr*-G4-1 sequence | 5’-CGGTGAGAGGGGAGGGCTGGAGCGGGCGGGGGCCGGCGGGTGGG -3’; |
| *Lhcgr*-G4-2 sequence | 5’GGCAGGCCGAGGGGCGGGCAGAGGGTACGGGCGGGCCCCCCGGG -3’; |

**Table S3 The primers used for ChIP-qPCR.**

| **Positions in gene promoter** | **Primer** |
| --- | --- |
| Mouse *Lhcgr*-CGI-I | F: 5’- GCTCTAGTCTCAAGCCCAGA -3’  R: 5’- AGGCCCTGGTTCTAAAGCTT -3’ |
| Mouse *Lhcgr*-CGI-II (G4) | F: 5’- TAGGGAAACAGCAATGGGGT -3’  R: 5’- GAGTGTGAGCTCCGGTGG -3’ |
| Mouse *Cyp19a1*-(-1652~ -1412) | F: 5’- CCCTACTCCACGGCTAAGAG -3’  R: 5’- TGTGGATTCCCTGATGTGCT -3’ |
| Mouse *Cyp19a1*-(-1436~ -1286) | F: 5’- CTGCAGCACATCAGGGAATC -3’  R: 5’- AGATGACCCACTCCAAGCTT -3’ |
| Mouse *Cyp19a1*-(-1295~ -1047) | F: 5’- TGGGTCATCTGCCATTGGAA -3’  R: 5’- GTGATGACTTGTGCCCTGTG -3’ |
| Mouse *Cyp19a1*-(-1066~ -882) | F: 5’- CACAGGGCACAAGTCATCAC -3’  R: 5’- GTCCTCTTCTGGTGTGTCTGA -3’ |
| Mouse *Cyp19a1*-(-902~ -740) | F: 5’- TCAGACACACCAGAAGAGGAC -3’  R: 5’- AGGGGCACGAATAAAGAGGG -3’ |
| Mouse *Cyp19a1*-(-759~ -537) | F: 5’- CCCTCTTTATTCGTGCCCCT -3’  R: 5’- TCCCGGACTGTACCCTTCTA -3’ |
| Mouse *Cyp19a1*-(-556~ -406) | F: 5’- TAGAAGGGTACAGTCCGGGA -3’  R: 5’- AAAAGGCTCCCGTACCCAAG -3’ |
| Mouse *Cyp19a1*-(-430~ -193) | F: 5’- TTAGACTTGGGTACGGGAGC -3’  R: 5’- CTTGGGAGGCTCAGGTTCTG -3’ |
| Mouse *Cyp19a1*-(-211~ 12) | F: 5’- AGAACCTGAGCCTCCCAAG -3’  R: 5’- GCGCTATTTGGCCTCAGAAG -3’ |
| Mouse *Rorα*-CGI-I | F: 5’- CCACCTTCCTCCTTCCAGAG -3’  R: 5’- GAGAAGAGGGAGGGAGGAGA -3’ |
| Mouse *Rorα*-CGI-II | F: 5’- TCTCCTCCCTCCCTCTTCTC -3’  R: 5’- AAAAGGAAAAGGAGACGGGC -3’ |

**Table S4** **The sequences of *Lhcgr* CGI-II, *Lhcgr* ΔG4-I, *Lhcgr* ΔG4-II and *Lhcgr* dΔG4** (5' KpnI highlighted in Yellow, 3' BglII highlighted in Cyan, the red letter indicates G-quadruplex)

| ***Lhcgr* promoter truncations** | **Sequence** |
| --- | --- |
| *Lhcgr* CGI-II | GGTACCGGCCACAGTCCCAGGTCAAGGAGAACAGGGACAGGCGGTGAGAGGGGAGGGCTGGAGCGGGCGGGGGCCGGCGGGTGGGAAGGCAGGCCGAGGGGCGGGCAGAGGGTACGGGCGGGCCCCCCGGGCGGTCCAGCATACTGGCCTAGCCACCGGAGCTCACACTCAGGCTGGCGGGCCAGATCT |
| *Lhcgr* ΔG4-I: | GGTACCGGCCACAGTCCCAGGTCAAGGAGAACAGGGACAGGCGGTGAGAGGGGATGGGAAGGCAGGCCGAGGGGCGGGCAGAGGGTACGGGCGGGCCCCCCGGGCGGTCCAGCATACTGGCCTAGCCACCGGAGCTCACACTCAGGCTGGCGGGCCAGATCT |
| *Lhcgr* ΔG4-II | GGTACCGGCCACAGTCCCAGGTCAAGGAGAACAGGGACAGGCGGTGAGAGGGGAGGGCTGGAGCGGGCGGGGGCCGGCGGGTGGGAAGGCAGGCCGACGGGCCCCCCGGGCGGTCCAGCATACTGGCCTAGCCACCGGAGCTCACACTCAGGCTGGCGGGCCAGATCT |
| *Lhcgr* dΔG4 | GGTACCGGCCACAGTCCCAGGTCAAGGAGAACAGGGACAGGCGGTGAGAGGGGATGGGAAGGCAGGCCGACGGGCCCCCCGGGCGGTCCAGCATACTGGCCTAGCCACCGGAGCTCACACTCAGGCTGGCGGGCCAGATCT |

**Table S5 The primers used for PCR cloning in luciferase assay (the underline indicates the restriction sites).**

| **PCR cloning** | **Primer** |
| --- | --- |
| *Lhcgr*-WT (-1558~ 88) | F: 5’- GGGGTACCGAGACACAGCATAAACCCGG -3’;  R: 5’- GAAGATCTGGCCCGCCAGCCTGAGTGTGAG -3’ |
| *Lhcgr*-ΔCGI-I (-1324~88) | F: 5’- GGGGTACCCTGAAAGGGGAAAGCAAAATC -3’;  R: 5’- GAAGATCTCGCCAGCCTGAGTGTGAG -3’ |
| *Lhcgr*-ΔCGI-II (-1558~-57) | F: 5’- GGGGTACCGAGACACAGCATAAACCCGG -3’;  R: 5’- GAAGATCTTGTCCCTGTTCTCCTTGACC -3’ |
| *Lhcgr*-Cyp19a1(-1599~-27) | F: 5’- GGGGTACCTCCCAGCCCTCATGGCAG -3’;  R: 5’- GAAGATCTTTCTATGGGAAGAAAGCAGT -3 |
| *Rorα*-WT ( -1422~-1) | F: 5’- GGGGTACCCGCAAAGTACAATGTCTCCCCA -3’;  R: 5’- GAAGATCT ATCGGCGGAGATGGCGAG -3’ |
