# Supplementary figures and images for: MeCP2 duplication causes hyperandrogenism by upregulating LHCGR and downregulating RORα
Source: Cell Death Dis. 2021 Oct 25;12(11):999. doi: 10.1038/s41419-021-04277-4 (PMC8545957; doi:10.1038/s41419-021-04277-4)

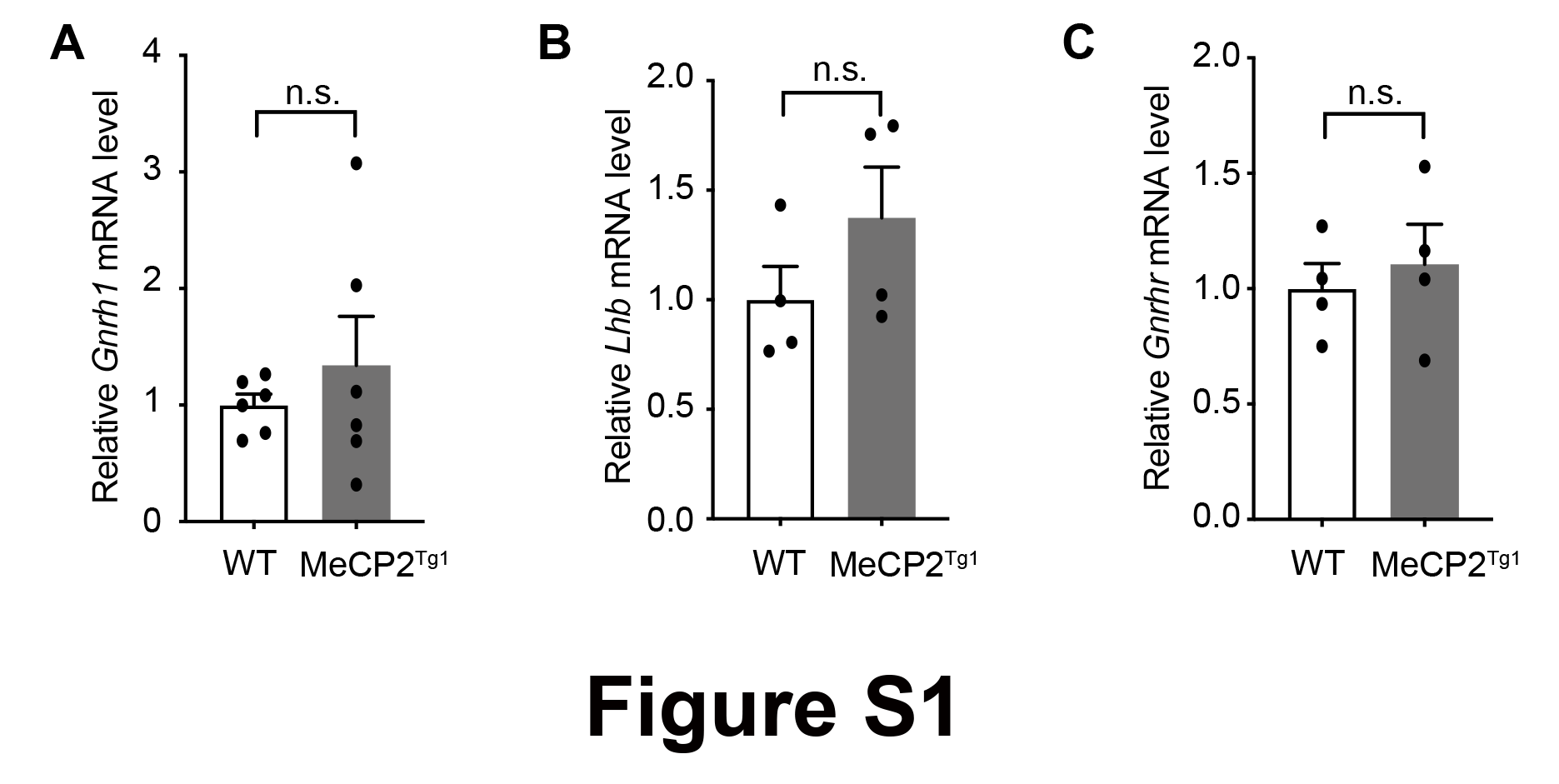

Supplement: Supplementary file 2 — Figure S1 [file 41419_2021_4277_MOESM2_ESM.tif]

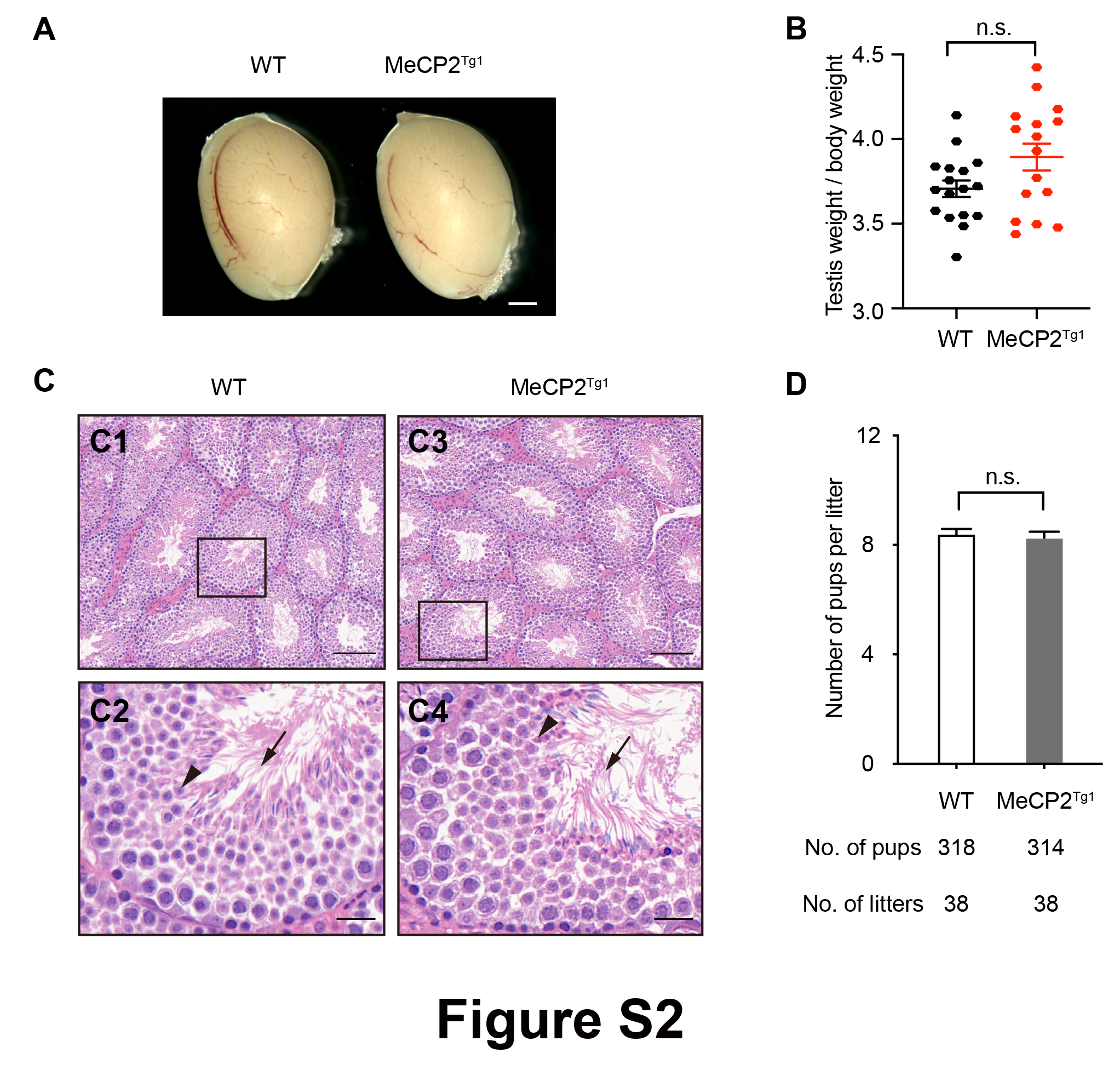

Supplement: Supplementary file 3 — Figure S2 [file 41419_2021_4277_MOESM3_ESM.tif]

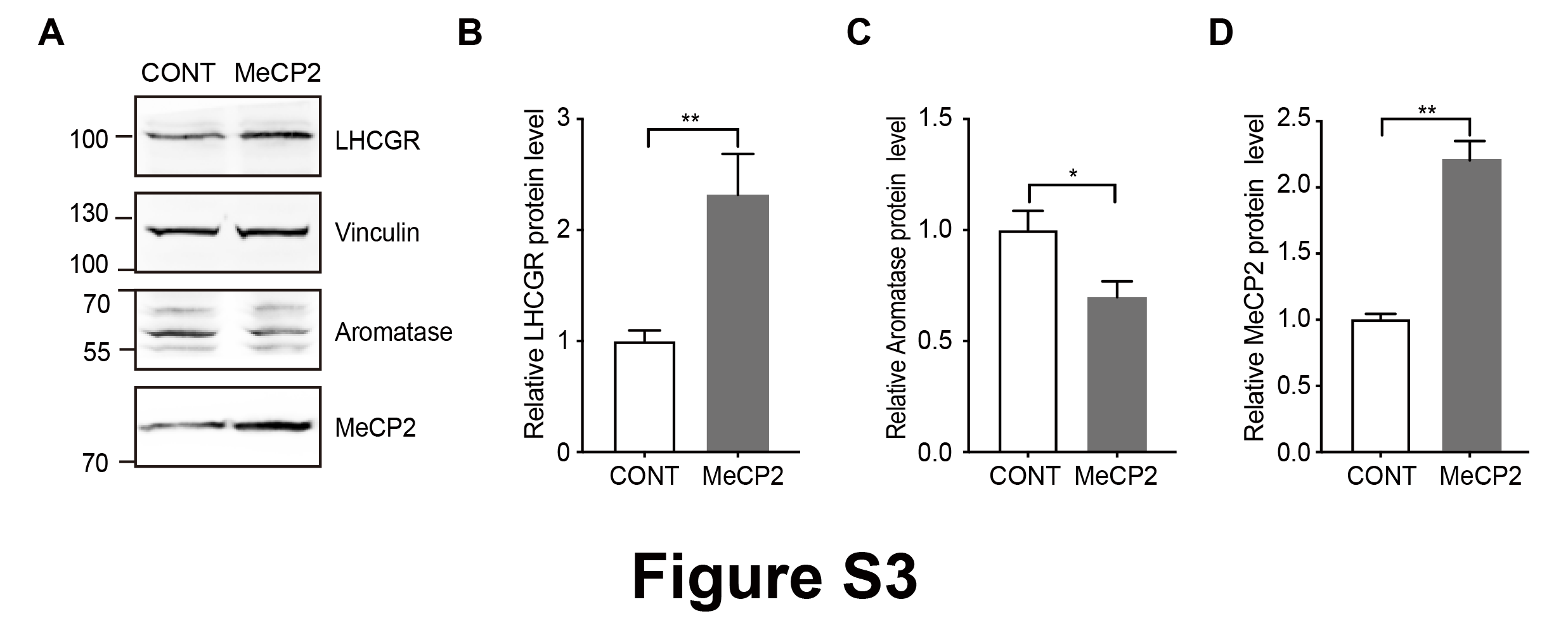

Supplement: Supplementary file 4 — Figure S3 [file 41419_2021_4277_MOESM4_ESM.tif]

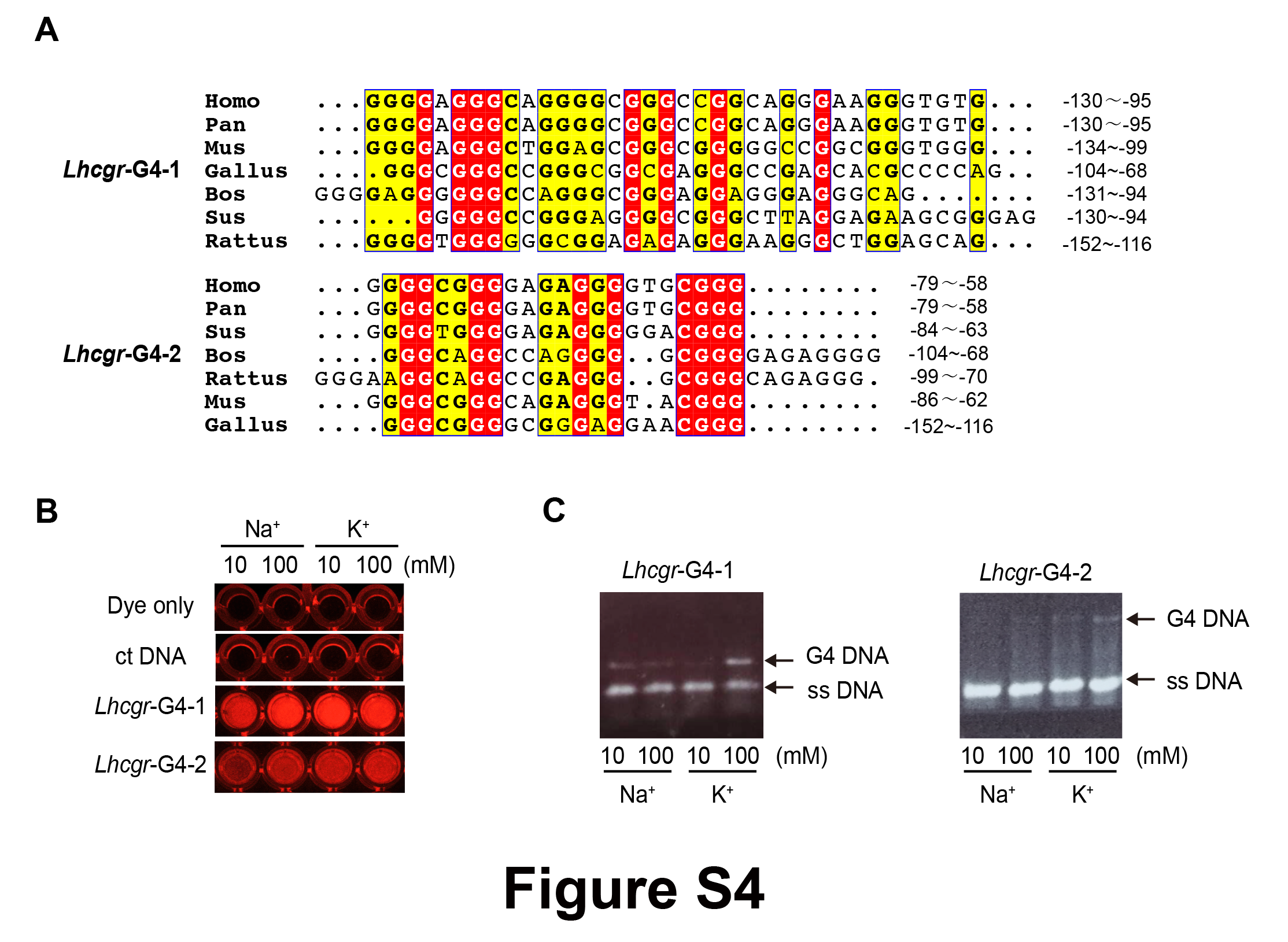

Supplement: Supplementary file 5 — Figure S4 [file 41419_2021_4277_MOESM5_ESM.tif]

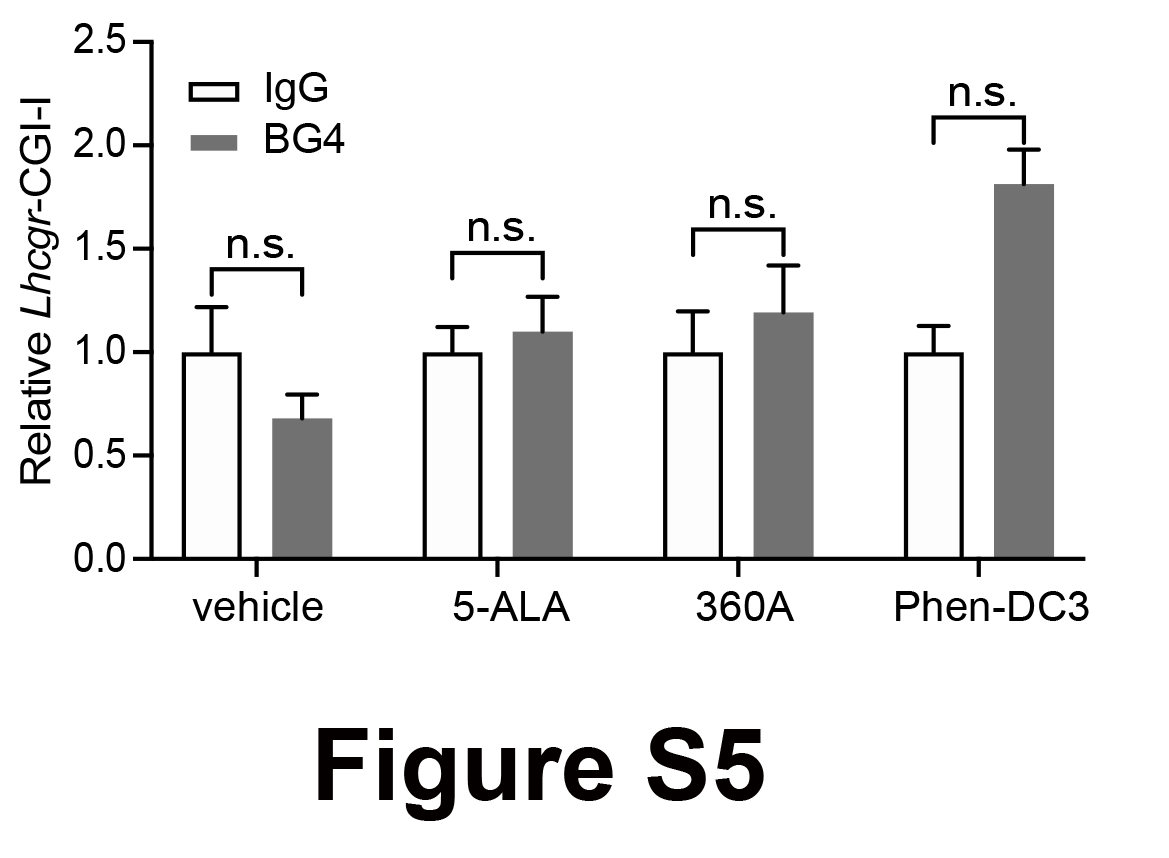

Supplement: Supplementary file 6 — Figure S5 [file 41419_2021_4277_MOESM6_ESM.tif]

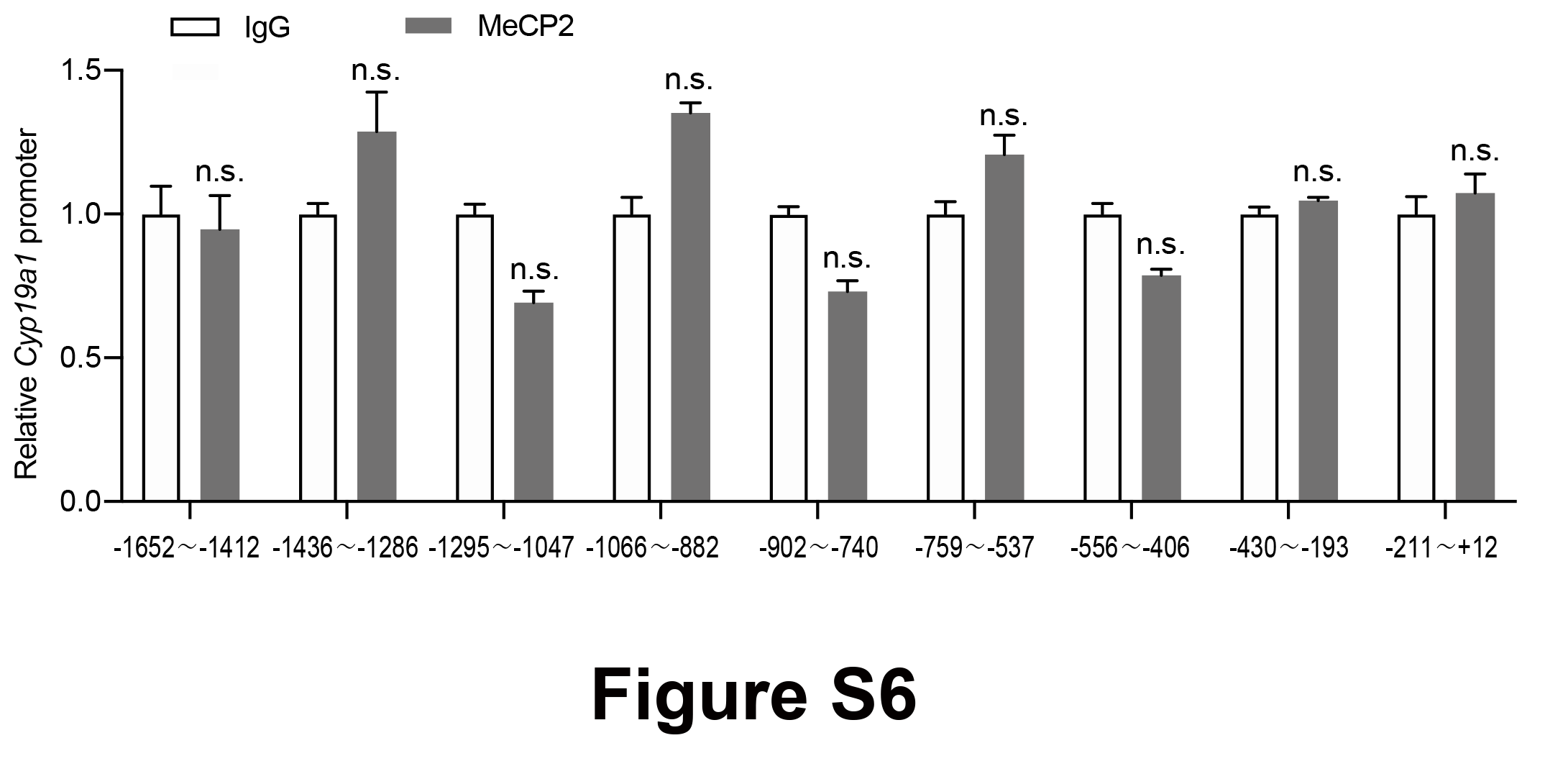

Supplement: Supplementary file 7 — Figure S6 [file 41419_2021_4277_MOESM7_ESM.tif]

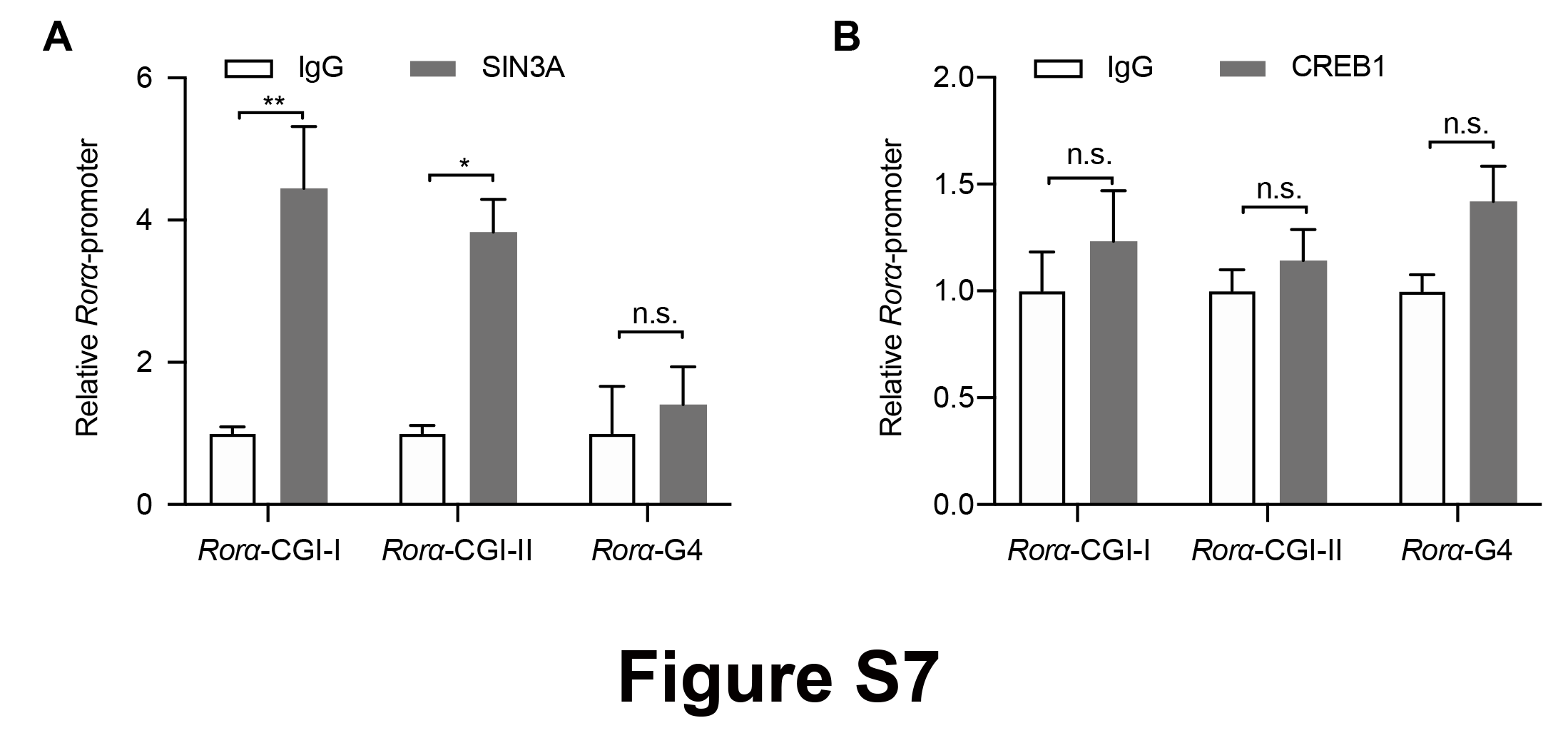

Supplement: Supplementary file 8 — Figure S7 [file 41419_2021_4277_MOESM8_ESM.tif]

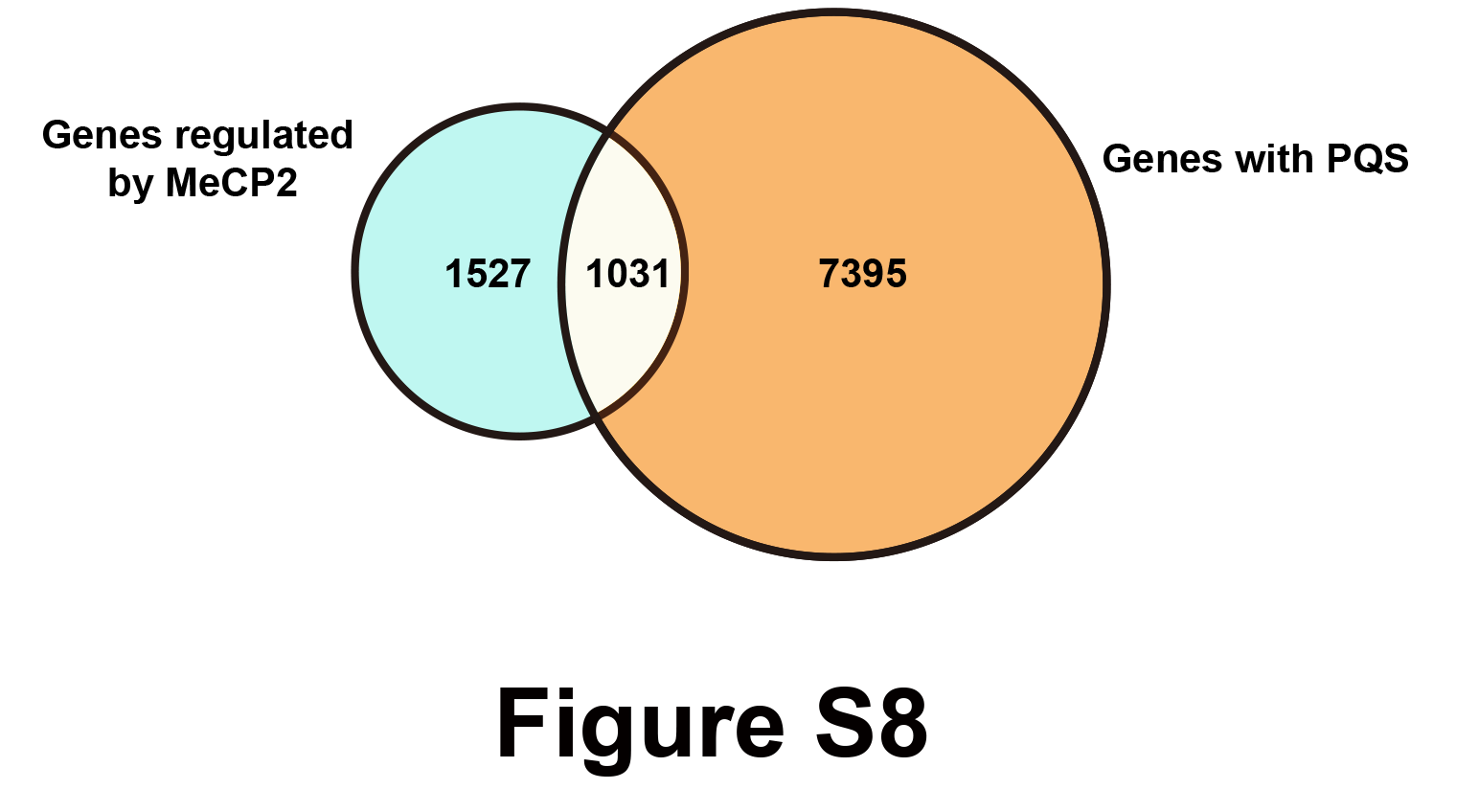

Supplement: Supplementary file 9 — Figure S8 [file 41419_2021_4277_MOESM9_ESM.tif]
